# *Insilico* generation of novel ligands for the inhibition of SARS-CoV-2 main protease (3CL^pro^) using deep learning

**DOI:** 10.3389/fmicb.2023.1194794

**Published:** 2023-06-23

**Authors:** Prejwal Prabhakaran, Ananda Vardhan Hebbani, Soumya V. Menon, Biswaranjan Paital, Sneha Murmu, Sunil Kumar, Mahender Kumar Singh, Dipak Kumar Sahoo, Padma Priya Dharmavaram Desai

**Affiliations:** ^1^Department of Biotechnology, New Horizon College of Engineering, Bangalore, India; ^2^Faculty of Biology, Albert-Ludwigs-Universität Freiburg, Freiburg im Breisgau, Germany; ^3^Department of Biochemistry, Indian Academy Degree College (Autonomous), Bangalore, India; ^4^Department of Chemistry and Biochemistry, School of Sciences, Jain (Deemed-to-be) University, Bangalore, India; ^5^Redox Regulation Laboratory, Department of Zoology, College of Basic Science and Humanities, Odisha University of Agriculture and Technology, Bhubaneswar, India; ^6^ICAR-Indian Agricultural Statistics Research Institute, PUSA, New Delhi, India; ^7^DBT-National Brain Research Centre, Gurugram, India; ^8^Department of Veterinary Clinical Sciences, College of Veterinary Medicine, Iowa State University, Ames, IA, United States; ^9^Department of Basic Sciences, New Horizon College of Engineering, Bangalore, India

**Keywords:** SARS-CoV-2, recurrent neural network, deep learning, 3CL^pro^, admet

## Abstract

The recent emergence of novel severe acute respiratory syndrome coronavirus 2 (SARS-CoV-2) causing the coronavirus disease (COVID-19) has become a global public health crisis, and a crucial need exists for rapid identification and development of novel therapeutic interventions. In this study, a recurrent neural network (RNN) is trained and optimized to produce novel ligands that could serve as potential inhibitors to the SARS-CoV-2 viral protease: 3 chymotrypsin-like protease (3CL^pro^). Structure-based virtual screening was performed through molecular docking, ADMET profiling, and predictions of various molecular properties were done to evaluate the toxicity and drug-likeness of the generated novel ligands. The properties of the generated ligands were also compared with current drugs under various phases of clinical trials to assess the efficacy of the novel ligands. Twenty novel ligands were selected that exhibited good drug-likeness properties, with most ligands conforming to Lipinski’s rule of 5, high binding affinity (highest binding affinity: −9.4 kcal/mol), and promising ADMET profile. Additionally, the generated ligands complexed with 3CL^pro^ were found to be stable based on the results of molecular dynamics simulation studies conducted over a 100 ns period. Overall, the findings offer a promising avenue for the rapid identification and development of effective therapeutic interventions to treat COVID-19.

## 1. Introduction

Coronavirus disease (COVID-19) caused by severe acute respiratory syndrome coronavirus 2 (SARS-CoV-2) has become a global public health crisis. Vaccines saved many lives despite numerous clinical trials for medicines against SAR-CoV-2 is under process (World Health Organization, 2020; Das et al., 2023). With nearly 765 million cases and 6.9 million deaths worldwide as of 3rd May 2023,^1^ there exists a vital need to identify or develop novel therapeutic interventions. Various studies have shown promising results in using repurposed drugs (reusing existing approved drugs for new medical indications) to inhibit the virus at different target sites (Elmezayen et al., 2020; Sarma et al., 2020). Among the target sites being considered, the 3-Chymotrypsin-like protease (3CL^pro^), is hypothesized to be a crucial target for the development of drugs (Khan et al., 2020; Tahir ul Qamar et al., 2020). 3CL^pro^ is responsible for the cleavage of polyproteins to produce non-structural proteins essential for viral replication (Elmezayen et al., 2020). Therefore, targeting 3CL^pro^ can inhibit the maturation and replication of the virus. 3-Chymotrypsin-like protease (3CL^pro^) and papain-like protease (PL^pro^) are essential enzymes in the peptide chain processing reaction. They cleave the C-terminus of the polypeptide chain at 11 sites and the N-terminus of the polypeptide chain at three sites. The cleavage products include structural proteins and some important non-structural proteins, such as RNA-dependent RNA polymerase (RdRp) and helicase. With more cleavage sites, 3CL^pro^ serves as an attractive non-structural protein for the development of drugs targeting SARS-CoV-2 (Li et al., 2020). The structure details are attached as a separate Supplementary file.

This protease contains several highly conserved substrate-binding sites within the active site of the enzyme, making it an attractive target for developing a diverse range of inhibitors. It is also exciting that the structures of 3CL^pro^ in SARS-CoV-2 and SARS-CoV differ by only 12 amino acids with comparable ligand binding efficiency (Macchiagodena et al., 2020). The 3-D structure and other details of the protease are attached as a PDBfile (RCSB, 2022). Jin et al. (2020) utilized the SARS-CoV2-PPC (protease pharmacophore clusters) to identify six principal protease flexible confirmations and active sites. The diverse druggable environments of the PPCs were explained by the presence of different sets of PPC consensus anchors in various PPCs, which affirmed the functionality of the PPCs. When a compound is present in a PPC, its protease binding affinities improve with an increasing number of occupied anchors, leading to a greater number of interactions (Pathak et al., 2021). The 3D crystalline structure of 3CL^pro^ was submitted to Protein Data Bank (PDB) in January 2020 under the PDB ID: 6 LU7 (Jin et al., 2020), and it was complexed with an N3 inhibitor. Thus, the active site of the N3 inhibitor could be chosen as the site for designing ligands that can potentially inhibit the activity of the protease (Corbeil et al., 2012; Paital et al., 2022).

During this period of a global pandemic, the drug discovery and development process must be accelerated, but one of the greatest impediments to this is the lead discovery process (Kadurin et al., 2017). To combat this issue, *in-silico* methods such as deep learning have emerged as a promising alternative, offering the potential to not only reduce costs but also significantly compress the timeline (Paital et al., 2015; Jin et al., 2020). These models can learn to generate new data that closely resembles the training data by extracting high-level features from the data (Kadurin et al., 2017). Deep learning has been successfully applied to generate novel molecules (Prykhodko et al., 2019) and has been reported to produce effective lead candidates in very little time (Gupta et al., 2017; Vanhaelen et al., 2017; Zhavoronkov et al., 2019).

In this study, a deep learning model based on a Recurrent Neural Network (RNN) was used to generate new ligands that could potentially act as inhibitors of 3CL^pro^. RNNs are highly effective in modeling sequential data with a temporal relationship, where each data point depends on the previous one. In this case, the RNN was trained on chemical molecules represented as SMILES strings. The model learns the relationship between each ASCII character and its temporal dependence in the input SMILES strings and predicts the ASCII character in the SMILES string based on the previous characters. A Long Short Term Memory (LSTM) network was specifically selected, as vanilla RNNs suffer from the vanishing gradient problem, where the gradient becomes smaller and smaller for large sequences of data (Menon, 2022).

Molecular docking was performed by virtual screening to identify the best hits against the viral protease. Evaluation of the molecular properties of the ligands and absorption, distribution, metabolism, excretion, and toxicity (ADMET) analysis were performed to study the biological activity and pharmacokinetic properties of the generated ligands. Additionally, molecular dynamic (MD) simulation was employed to investigate the stability and interaction of the ligand-protease complex for a duration of 100 nanoseconds. Finally, the properties of the generated novel ligands were compared to drugs that are currently in clinical trials as a therapeutic intervention for COVID-19. This study evaluates the effectiveness and potential of the newly generated ligands in inhibiting the main viral protease (3CL^pro^) of SARS-CoV-2.

## 2. Materials and methods

### 2.1. Technical implementation

The RNN was implemented using Tensorflow (v2.0^2^) and Keras (v2.3^3^) in Python (v3.7^4^) and RDkit^5^ was used for the processing of the molecules.

### 2.2. Recurrent neural network

To accelerate the synthesis of potential inhibitors against 3CL^pro^, a transfer learning approach was applied. Transfer learning is a machine learning technique where a pre-trained model is used as a starting point for training a new model with a similar task or domain. This allows the model to leverage the knowledge and experience gained from the pre-training to adapt to the new data and tasks more quickly and efficiently. In other words, transfer learning allows for faster and more accurate model development by building on top of previously learned representations.

Here, a publicly available model named LSTM_Chem (License: CC BY-NC-ND 4.0) was used (Gupta et al., 2017). The model consists of two LSTM layers with a 256-sized hidden state vector. It is regularized, having dropout layers. The two layers are followed by a final dense output layer with the softmax activation function. The model input is a bit array sequence of the molecule in the simplified molecular-input line-entry system (SMILES) format. This model was initially trained to produce novel TRPM8 inhibitors.

### 2.3. Dataset curation

For the LSTM_Chem model to generate potential inhibitors for 3CL^pro^, it was necessary to retrain and optimize the model on a ligand dataset that exhibits a certain degree of activity against 3CL^pro^. The training process involved two stages with distinct datasets. The first stage-trained the model on a training dataset to learn the latent space features of chemical molecules. The second stage involved fine-tuning the model using a separate dataset to enable it to generate ligands that possess the chemical features of protease inhibitors for COVID-19.

**Figure 1:**
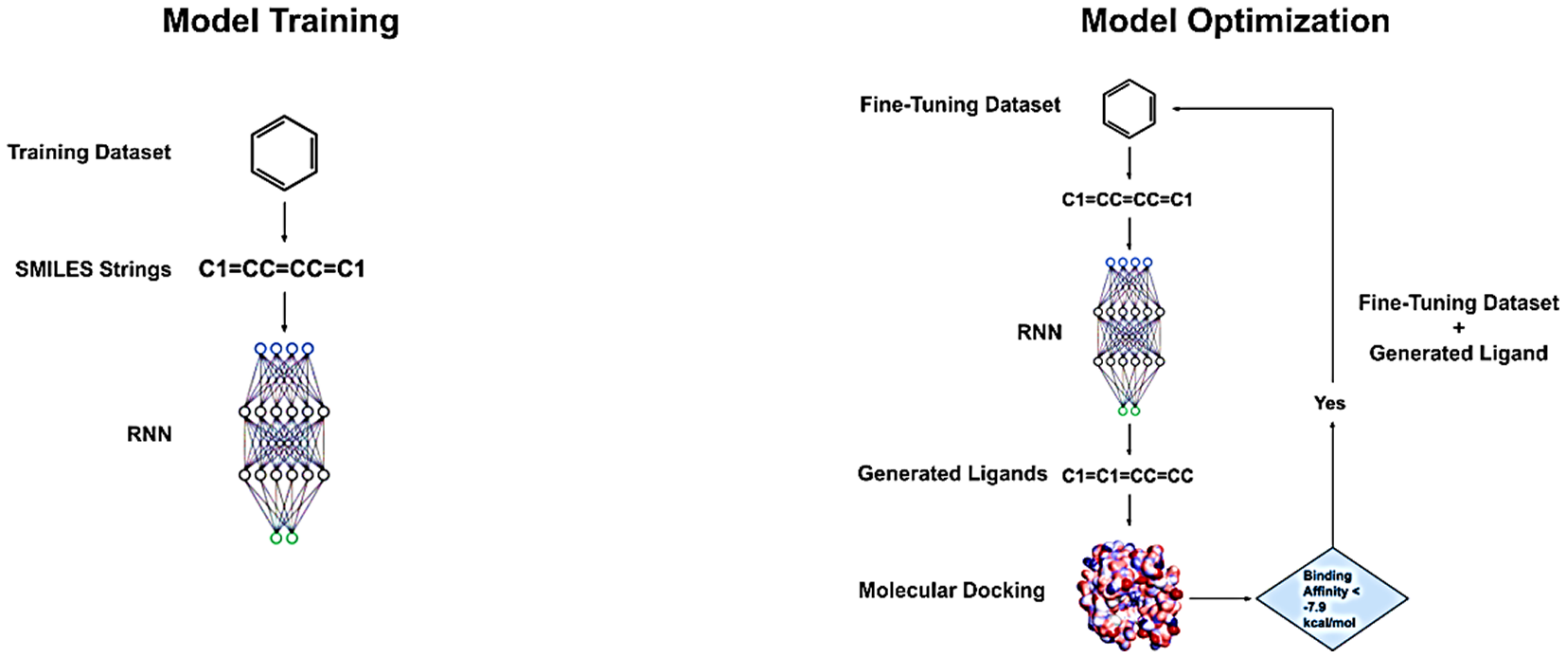
Model training and optimization.

The training dataset consists of a large volume of diverse ligands from which the RNN learns to produce valid ligands with high accuracy (Figure 1). The dataset was obtained from ChEMBL22^6^ and contained 556,134 SMILES strings, which were processed to remove duplicates, salts, and stereochemical information, resulting in a collection of unique ligands. Furthermore, only SMILES strings that had lengths between 34 and 74 tokens were retained, leading to a final size of 439,217 SMILES strings. This methodology was chosen following the work done by Gupta et al. (2017). The SMILES string length was constrained as having very long strings would result in the vanishing gradient problem, and the network would not learn anything. Although LSTMs are good at tackling the vanishing gradient, they are not completely immune to it (Moret et al., 2019). Additionally, the LSTM_Chem model accepts a bit array sequence as input, which was obtained by converting the SMILES strings using the Morgan algorithm in RDKit Open-source cheminformatics; (Open-Source Cheminformatics, see Footnote 5). The Morgan algorithm is a graph relaxation algorithm used for molecule canonicalization, which assigns a unique identifier to a molecule regardless of its representation. However, the Morgan algorithm has known issues that can result in noncanonical atom orderings and can be problematic when used with large molecules such as proteins. Therefore, restricting the length of the SMILES strings to fall within a range of 34–74 tokens limits the size of the molecules to small ligands and reduces the likelihood of encountering issues with the Morgan algorithm (Schneider et al., 2015; Moret et al., 2019; Schneider, 2019).

To create a dataset for fine-tuning the model, 845 drugs undergoing clinical trials and drugs that demonstrated activity in different biological assays for COVID-19 were collected from PubChem.^7^ This dataset included compounds that showed activity against not only 3CL^pro^ but other drug targets of SAR-CoV-2 as well. In PubChem BioAssay, “PUBCHEM_ACTIVITY_OUTCOME” is a column that reports the outcome of a specific assay run for a given compound. It describes whether the tested compound showed activity (i.e., produced a measurable effect) against the target of interest or not. Only compounds that were termed “active” were selected. The ligand structures were retrieved in SDF format using compound IDs, via PUG_REST, an application program interface (API) for accessing PubChem (Kim et al., 2018), and SMILES strings were generated using the *MolToSmiles* function from RDKit. This dataset was preprocessed similarly to the training dataset, resulting in 639 ligands that were used for fine-tuning the model. However, unlike the training dataset, no restriction was set on the string length of the SMILES in the fine-tuning dataset.

### 2.4. Training and optimization

The model underwent training for 50 epochs on the training dataset, followed by 25 epochs of fine-tuning on the fine-tuning dataset. During fine-tuning, 1,000 SMILES strings were generated and evaluated for their validity. The validated ligands were then subjected to docking onto 3CL^pro^. The ligands that exhibited a binding affinity greater than that of the native ligands were incorporated back into the fine-tuning dataset. The fine-tuning dataset underwent preprocessing and shuffling, with this entire fine-tuning process being repeated for three cycles. This process of adding validated ligands with high-binding affinity back into the dataset and repeating the fine-tuning process is a way to iteratively improve the performance of the model and helps it identify the characteristics of ligands that contribute to their strong binding affinity with 3CL^pro^. A schematic overview of the model training and optimization process is provided in Figure 1.

### 2.5. Virtual screening

Virtual screening involves docking ligand libraries to a target macromolecule to discover a lead that would confer a biological function. The virtual screening was done using AutoDock Vina in PyRx (Dallakyan and Olson, 2014).

The generated ligands were converted from SMILES to SDF using openBabel-GUI (O’Boyle et al., 2011). To obtain the lowest free energy of the ligand, the Merck molecular force field (mmff94) parameter was used in PyRx. Finally, the ligands were converted to PDBQT format, preparing the ligand for molecular docking (Morris et al., 1998; Huey et al., 2007).

The 3D crystalline structure of SARS-CoV-2 main protease or 3CL^pro^ (PDB: 6 LU7) was obtained from PDB^8^; this served as the target for docking. The target was prepared by removing the native ligand present (N3 Inhibitor) and water molecules using Biovia Discovery Studio (Biovia, 2017).

The native ligand was docked onto the target molecule, and the binding affinity was found to be −7.9 kcal/mol. The amino acid residues involved in binding with the native ligand were obtained using the 2D structure in Biovia Discovery Studio. The amino acid residues are Thr24, Thr26, Phe140, Asn142, Gly143, Cys145, His163, His164, Glu166, His172. The grid box was then positioned over the binding site (center: *x* = −10.606, *y* = 17.214, *z* = 64.716, total size: *x* = 24.074 Å, *y* = 24.134Å, *z* = 19.174Å).

Further post-docking analysis and visualization of the ligand-target complex were carried out in Biovia Discovery Studio (Biovia, 2017).

### 2.6. Evaluation of molecular properties

To evaluate the molecular properties of the ligands, an online tool, Molinspiration^9^ was used by uploading the ligands in SMILES format. Molinspiration also provides bioactivity scores for drug targets such as Ion channel modulators, GPCR (G protein-coupled receptor) ligands, kinase inhibitors, nuclear receptor inhibitors, protease inhibitors, and enzyme inhibitors. The bioactivity score of Molinspiration is calculated by a machine learning-based model that predicts the probability of a molecule being active against a particular target. The model is trained on a large database of known active and inactive compounds and uses various molecular descriptors, such as physicochemical properties and substructure information, to make predictions. These bioactivity scores provide an additional metric for evaluating the drug-like properties of the ligands (Vardhan and Sahoo, 2020). Another online tool Molsoft^10^ was used to evaluate the drug-likeness score of the ligands (Prabhavathi et al., 2020).

### 2.7. Evaluation of ADMET profile

The absorption, distribution, metabolism, elimination, and toxicity (ADMET) are some of the important pharmacokinetic properties that must be evaluated. An online tool called admetSAR^11^ was used to obtain the ADMET profile of the ligands (Yang et al., 2018). Some of the properties calculated include ames mutagenesis, blood-brain barrier penetration, BSEP inhibition, Caco-2, Carcinogenicity, cytochrome p450 substrate and inhibitors, glucocorticoid receptor binding, hepatotoxicity, human ether-a-go-go (hERG) inhibition, p-glycoprotein inhibitors and substrate, human intestinal absorption, and human oral bioavailability.

### 2.8. Curating the reference dataset

To evaluate the capability of the generated ligands as potential anti-COVID drugs, a reference dataset comprising 20 drugs currently in clinical trials for COVID-19 treatment was obtained from PubChem. This included drugs such as Remdesivir, Ritonavir, Galidesivir, etc. (For full list of drugs—Appendix A). The properties of the generated ligands were compared to those of the reference drugs to assess their potential as anti-COVID agents. This comprehensive comparison of the ligands and clinical trial drugs facilitates the assessment of the ligand’s properties.

### 2.9. Molecular dynamics simulations

Molecular Dynamics simulation is a sophisticated computational tool for predicting and analyzing the dynamic behavior of molecules (Verdonk et al., 2003; Radinnurafiqah et al., 2016; Girdhar et al., 2019; Choubey et al., 2022; Mishra et al., 2022). The stabilities of six selected protein-ligand complexes were assessed using GROMACS 2021 package through Molecular Dynamics (MD) simulations (Van Der Spoel et al., 2005). The complexes included SARS_COV2_MOL_1, SARS_COV2_MOL_3, SARS_COV2_MOL_9, SARS_COV2_MOL_10, SARS_COV2_MOL_17, and SARS_COV2_MOL_20. The ligand topology parameter for CHARMM forcefield (Vanommeslaeghe et al., 2010) was created using the CGenFF server.^12^ A cubic box of TIP3P water models was used to solvate all the complexes. To maintain the periodic boundary conditions, the distance between the protein and the box edge was kept at 1 nm. The systems were neutralized by adding 0.15 M NaCl. Energy minimization was performed using the steepest descent method followed by the conjugate gradient method with maximum number of minimization 50,000 per alogorithm. The Particle Mesh Ewald (PME) method was employed to calculate long-range interactions (Abraham and Gready, 2011). The first phase of equilibration was carried out with an NVT ensemble, where the temperature was equilibrated using 50,000 iterations of 2 fs each. In the second phase, the pressure was equilibrated at 300 K with an NPT ensemble using Parrinello-Rahman, a pressure coupling method. The temperature inside the system was regulated using V-rescale, a modified Berendsen thermostat. Finally, a production run of 100 ns was established to gain insights into the dynamic behavior of the complex.

### 2.10. Trajectory analysis

The obtained trajectories after the MD simulations were analyzed for calculations such as root mean square deviation (RMSD), root mean square fluctuation (RMSF), radius of gyration (Rg), solvent accessible surface area (SASA), and inter-molecular hydrogen bond using the in-built tools of the GROMACS package. To compute the RMSD in the protein backbone, the *rms* module of GROMACS was employed. RMSD of the ligands were also calculated using the same module, whereas the *rmsf* module was used to determine the RMSF in the atomic positions of the protein Cα backbone. In addition, modules like h-bond, gyrate, and SASA were used to calculate the number of hydrogen bonds, Rg, and SASA, respectively.

## 3. Results

A deep learning model called LSTM_Chem was trained, using transfer learning, to produce novel ligands that could inhibit 3CL^pro^, the main viral protease of SAR-CoV-2. The ligands’ ability to inhibit the protease is evaluated through molecular docking, ADMET analysis, and molecular dynamics simulation.

### 3.1. Selection of generated ligands

After the first stage of training on the training dataset, a final loss of 0.427 on the training set and 0.567 on the validation set (20% of data from the training dataset) was obtained. Additionally, the model had an accuracy of 82% in generating valid ligands, i.e., out of every 100 ligands the model produces, 82 are valid molecules.

**Figure 2:**
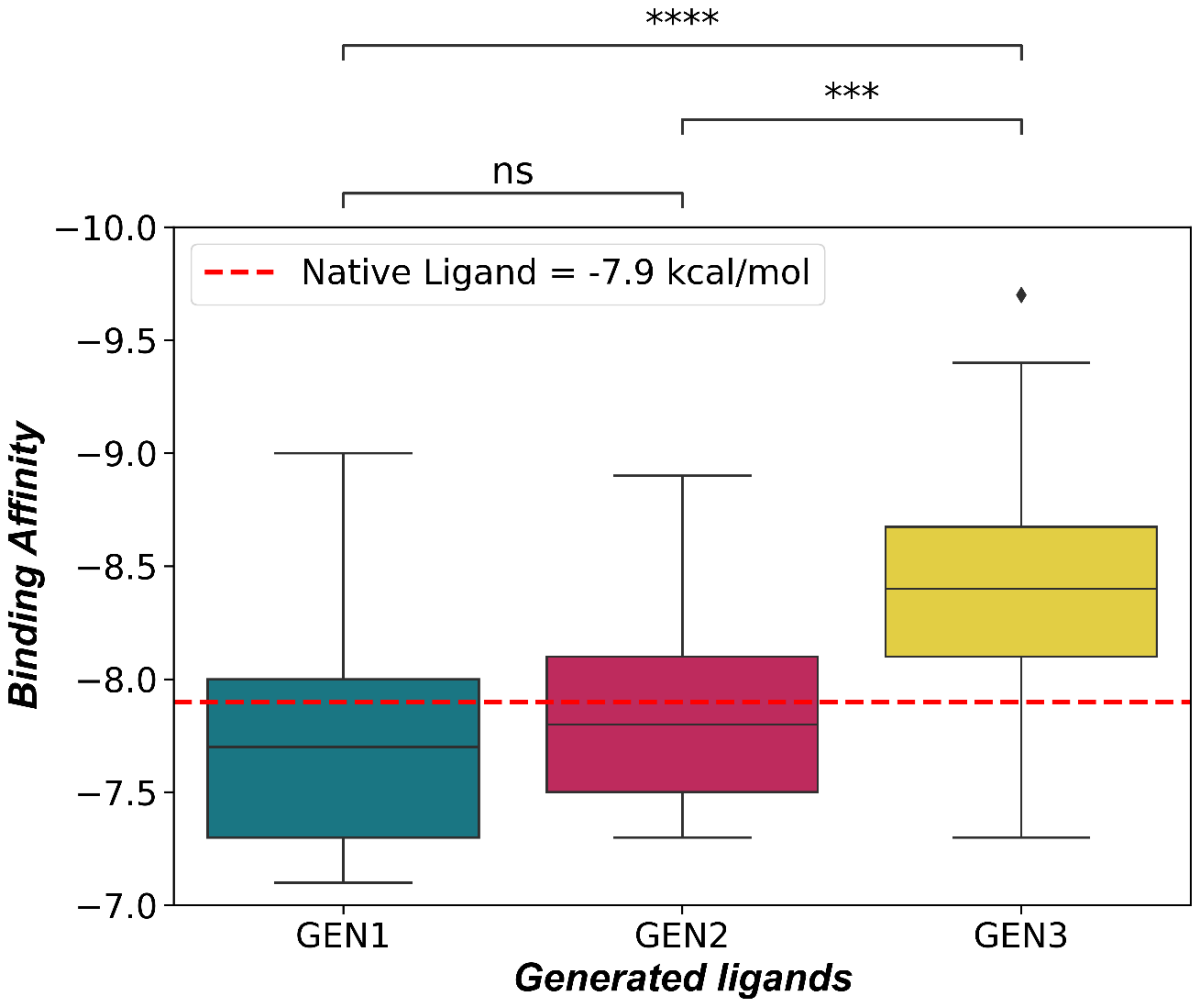
Binding affinity of generated ligands after 3 cycles of fine-tuning. Ligands generated after the 3rd cycle exhibit significantly better binding affinity to 3CL^pro^ (Mann–Whitney U Test, ***≥ *p* < 0.001, ****≥ *p* < 0.0001) than the previous generations.

The model then underwent three cycles of fine-tuning on the fine-tuning dataset, and after each cycle, the binding affinity of 30 randomly selected ligands was evaluated. Figure 2 depicts the distribution of the binding affinities across the three cycles, indicating that the 3rd generation of molecules had a significantly better binding affinity to 3CL^pro^ than the previous two generations (Mann Whitney U Test, *p* < 0.001). The average binding affinity for the 3rd generation was −8.406 ± 0.087 kcal/mol, and 26 (86.67%) of the ligands had a value higher than the binding affinity of the native Ligand (−7.9 kcal/mol).

After evaluating the binding affinity of 30 ligands generated by the model, the top 20 ligands were chosen for additional investigations. To confirm the novelty of these molecules, a search was conducted in the PubChem database, which did not yield any results for these ligands. Therefore, it can be inferred that the generated molecules are novel. The molecules were named SARS_COV2_MOL_1 - SARS_COV2_MOL_20 as an identifier.

### 3.2. Comparative analysis

Various molecular and ADMET properties of the generated ligands and drugs in the reference dataset were calculated and contrasted to assess the efficacy of the generated ligand to serve as a potential inhibitor to the 3CL^pro^ protease (Tables 1, 2).

**Table 1:** Comparison of Lipinski’s parameters between generated and reference ligands.

Property	% compliance with Ro5 (generated ligands)	Mean ± SD	% compliance with Ro5 (reference drugs)	Mean ± SD	% difference
MW ≤ 500	65	527.18 ± 29.55	70	434.44 ± 48.13	−5
HBD ≤ 5	95	3.0 ± 0.40	90	3.45 ± 0.42	5
HBA ≤ 10	85	8.55 ± 0.59	80	8.25 ± 0.81	5
NRB ≤ 10	60	10.8 ± 0.81	70	7.55 ± 1.13	−10
LogP ≤ 5	80	3.85 ± 0.41	85	2.61 ± 0.73	−5
TPSA ≤ 140	75	825.51 ± 709.55	60	120.01 ± 10.78	15

**Table 2:** Comparison of bioactivity scores between reference and generated ligands.

	Mean reference ligands	Reference ligands score > - 0.5 (%)	Mean generated ligands	Generated ligands score > - 0.5 (%)
Enzyme inhibitor	−0.15 ± 0.26	80	−0.35 ± 0.18	80
Ion channel modulator	−0.52 ± 0.25	75	−0.62 ± 0.23	65
Kinase inhibitor	−0.25 ± 0.21	50	−0.58 0 ± 0.20	65
GPCR ligand	−0.22 ± 0.25	80	−0.12 ± 0.15	85
Nuclear receptor inhibitor	−0.90 ± 0.26	45	−0.61 ± 0.21	65
Protease inhibitor	−0.21 ± 0.21	80	0.05 ± 0.11	90

#### 3.2.1. Assessment based on Lipinski’s rule

**Figure 3:**
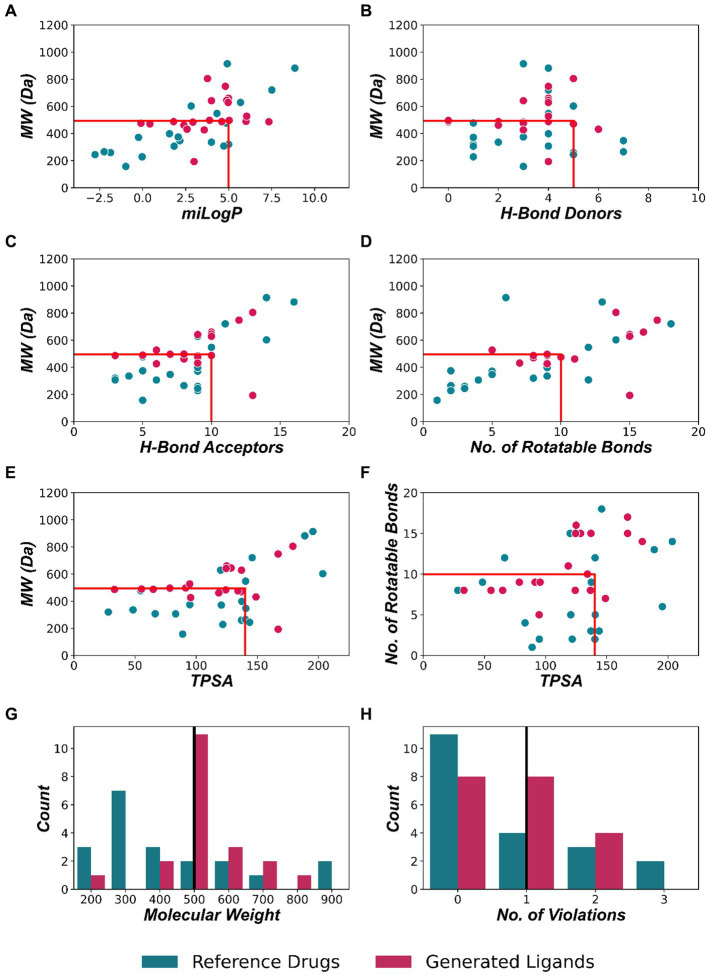
Comparative evaluation of structural properties between reference drugs and generated ligands based on Lipinski’s rule of 5. **(A)** Lipophilicity (miLogP), **(B)** number of H-Bond donors, **(C)** number of H-bond acceptors, **(D)** number of rotatable bonds, **(E)** total polar surface area is compared to the molecular weight (MW). **(F)** The total polar surface area (TPSA) vs. number of rotatable bonds. **(A–F)** The red box indicates the ligands that comply with Lipinski’s rule. **(G)** Distribution of molecular weight and the black line depicts the Lipinski’s criteria for MW < 500 Da. **(H)** Distribution of the number of violation to Lipinski’s rule. Having 1 or less violation of Lipinski’s criteria imply molecules with drug-like properties (depicted by the black line).

Lipinski’s rule of 5 provides a set of criteria to estimate the solubility and permeability of a ligand. This has become a crucial criterion for assessing the oral bioavailability of any drug during the drug development process. The criterion for oral activity is based on the molecular properties of drugs such as molecular weight (MW ≤ 500), partition coefficient (logP ≤ 5), hydrogen bond donors (HBD ≤ 5), hydrogen bond acceptors (HBA ≤ 10), and the number of rotatable bonds (NRB ≤ 10) (Lipinski et al., 1997). Table 1 and Figure 3 show the various molecular properties of generated and reference ligands plotted to assess their compliance with Lipinski’s Ro5.

According to Lipinski’s Rule, ligands having less than or equal to 1 violation of Lipinski’s criteria can be considered to have oral bioactivity. 16 (80%) of generated ligands exhibit 0 or 1 violation, and all ligands show less than or equal to 2 violations. 19 (95%) generated ligands have less than 5 H Donors, and 17 (85%) have less than 10 H Acceptors. Octanol-water partition coefficient or logP is used as a measure of molecular lipophilicity. Lipophilicity affects drug absorption, bioavailability, hydrophobic drug-receptor interactions, metabolism of molecules, as well as their toxicity. It is one of the key parameters that determine the drug-likeness of compounds (Amézqueta et al., 2020). 16 (80%) compounds among the generated ligands exhibit a LogP < 5.00.

For the oral bioavailability of compounds, the molecular weight of the compound should be ≤500 Da. 13 (65%) generated ligands and 14 (70%) ligands in the reference dataset were found to have a molecular weight less than 500. The average molecular weight among the generated ligands was found to be 527.176 ± 29.552 Da. Refer to Table 1 for additional information.

#### 3.2.2. Assessment based on bioactivity score

**Figure 4:**
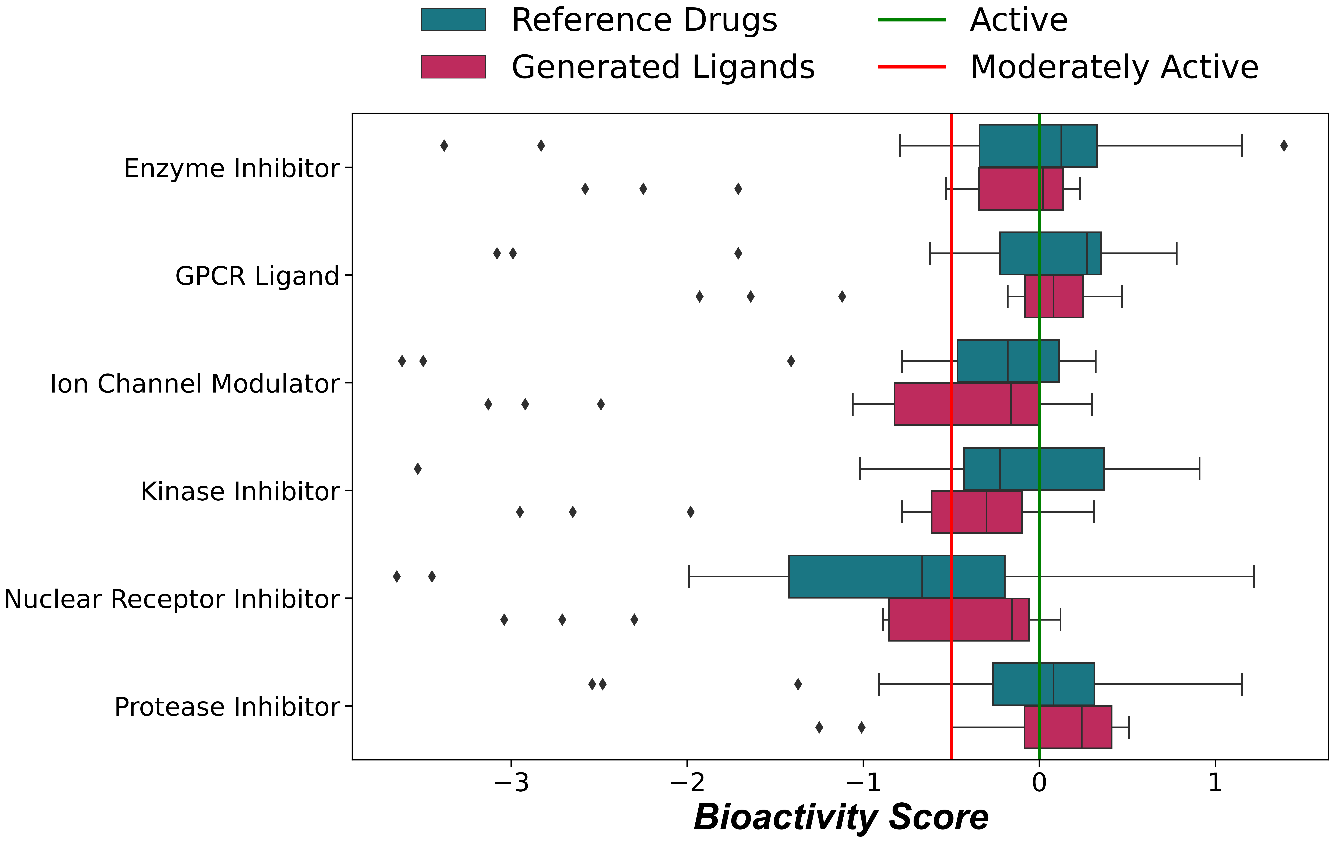
Distribution of bioactivity scores from Molinspiration between generated Ligands and reference drugs. The green line represents a bioactivity score of 0; ligands above this line are considered to be active, the red line represents a bioactivity score of −0.5, below which ligands are considered to be inactive; in the region between the red and green line, ligands are considered to be moderately active.

Molinspiration was used to obtain the bioactivity scores of the generated ligands and the reference drugs. Bioactivity here refers to a quantitative estimate of the compound’s potency and efficacy in inhibiting or activating various targets. Compounds with a bioactivity score of more than 0 are considered biologically active, while values between −0.50 and 0.00 are considered moderately active, and less than −0.50 are inactive (Khan et al., 2017). Figure 4 represents the distribution of the bioactivity score for the generated and reference ligands. It can be referred from Table 2, that the generated ligands and reference drugs have comparable bioactivity scores. 14 (70%) generated ligands show a bioactivity score greater than 0 as a protease inhibitor. This suggests that the ligands share structural characteristics with other protease inhibitors, indicating a high likelihood of their potential as protease inhibitors.

#### 3.2.3. Assessment based on docking

**Figure 5:**
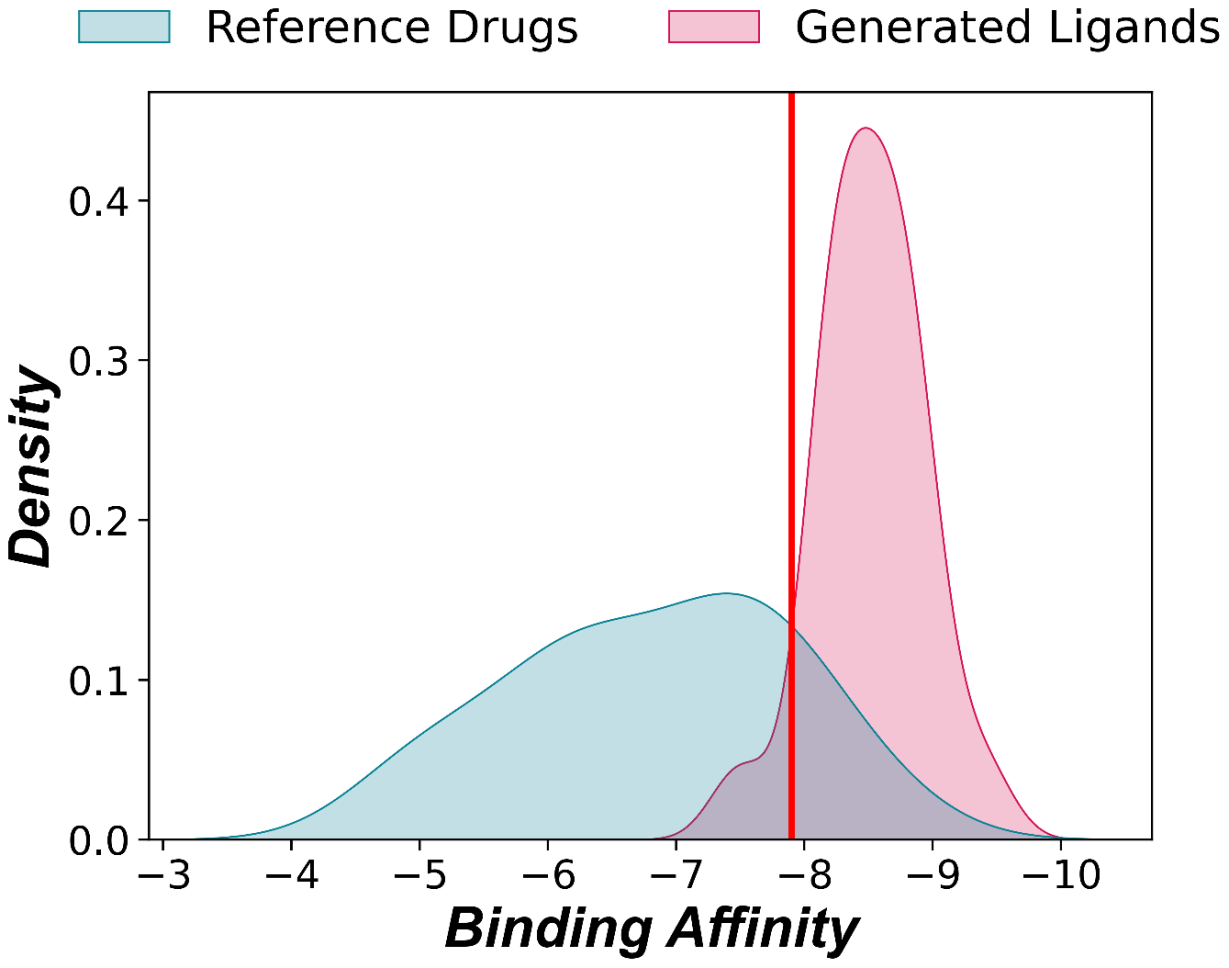
Distribution of binding affinities of generated and reference ligands to 3CL^pro^ (Structure: 6 LU7). The red line represents the binding affinity of the native ligand (−7.9 kcal/mol).

The generated ligands exhibit a strong binding affinity toward the target protease 3CL^pro^ (6 LU7), as evidenced by molecular docking results presented in Figure 5 and Appendix A. These results indicate that the binding affinities of the generated ligands are higher than that of the N3 inhibitor in the 6 LU7 structure of 3CL^pro^, which is −7.9 kcal/mol. On average, the generated ligands display a binding affinity of −8.515 ± 0.091 kcal/mol toward the target protease 3CL^pro^. The highest binding affinity among the generated ligand was −9.4 kcal/mol, and the lowest was −7.5 kcal/mol.

Among the reference drugs, Nafamostat had the highest binding affinity at −8.6 kcal/mol, while Fingolimod and Favipiravir had the lowest at −5.0 kcal/mol. The average binding affinity of the reference drugs was −6.81 ± 0.238, which is significantly lower than the binding affinity of the native ligand.

**Figure 6:**
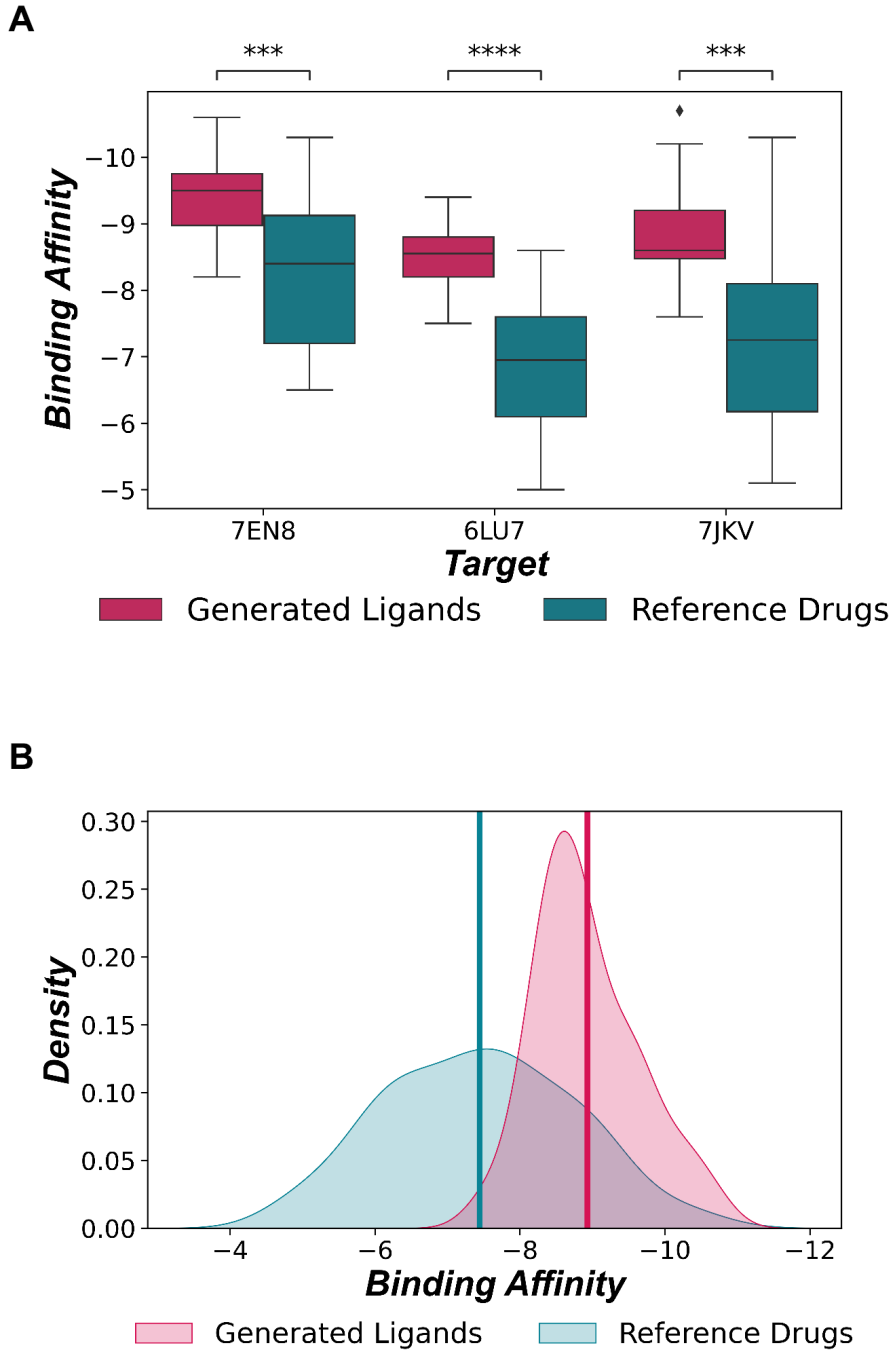
**(A)** Evaluation of binding affinities for three 3CL^pro^ PDB structures. The generated ligands show significantly better binding affinity than the reference drugs (Mann–Whitney U Test, ***≥ *p* < 0.001, ****≥ *p* < 0.0001). **(B)** Distribution of binding affinity across all three 3CL^pro^. The mean binding affinity of generated ligands (−8.928 ± 0.091 kcal/mol) and reference drugs (−7.441 ± 0.164 kcal/mol) are depicted by red and green lines, respectively.

The generated ligands were further evaluated for their binding energy with two additional SARS-CoV-2 3CL^pro^ structures, 7EN8, and 7JKV in the PDB database. The results showed that the binding affinities of the ligands to these structures were significantly better than the reference drugs (Mann Whitney U Test, *p* < 0.001) (Figure 6A). The average binding affinity of the generated ligands for all three structures was −8.928 ± 0.091 kcal/mol (Figure 6B).

#### 3.2.4. Assessment based on ADMET properties

**Figure 7:**
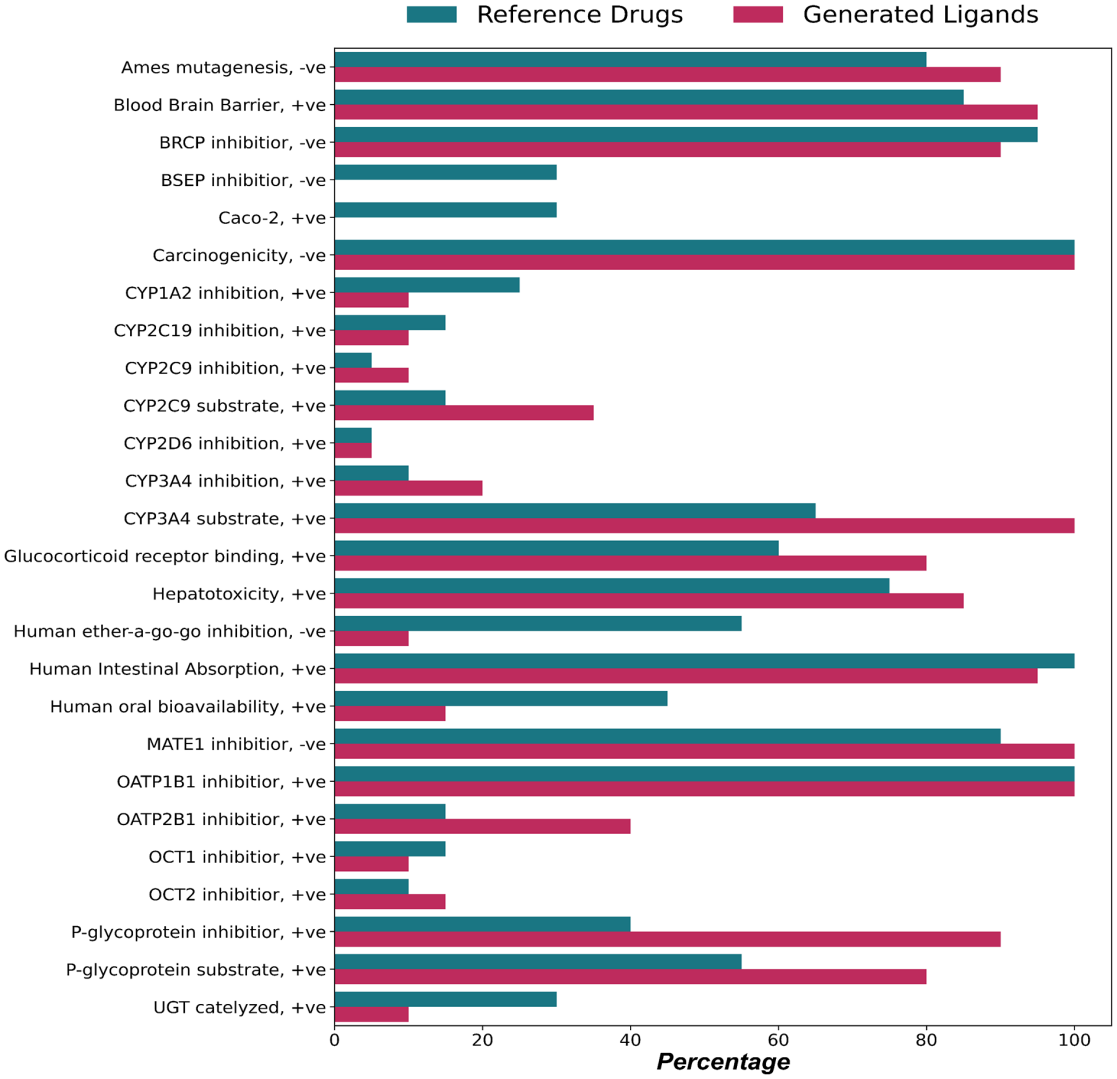
Absorption, distribution, metabolism, excretion, toxicity (ADMET) properties of generated and reference ligands.

To evaluate the pharmacokinetic properties of the reference and generated ligands admetSAR was used. Figure 7 provides an overview of the various ADMET properties assessed. The generated ligands exhibit a good degree of human intestinal absorption (95%) compared to the reference ligands (66.67%). Although the generated ligands show low oral bioavailability (15%) and none permeate through Caco-2 monolayer, from Lipinski’s Ro5 and TPSA predicted earlier (Figure 3), it can be inferred that the ligands will be absorbed effectively after administration.

P-glycoprotein (P-gp) is an efflux transporter found in various organs and it plays a vital role in the distribution of drugs. 16 (80%) of the generated ligands of the present study were found to be acting as substrates for P-gp. Cytochrome P450 is known to be one of the most important drug-metabolizing families of enzymes. Out of the 57 different CYP genes in the human body, it is established that only about a dozen gene products mediate most of the biotransformation reactions against foreign substances. 100% of the generated ligands were substrates for CYP3A4, which is responsible for the metabolism of nearly 50% of all drugs in clinical use (Zanger and Schwab, 2013), and 35 and 10% of the ligands were also CYP2C9 and CYP2D6 substrates, respectively. One of the major drug excretion routes is the renal organic cation transporters (OCT) (Yin and Wang, 2016), and inhibitors of OCT are known to cause renal toxicity leading to excess drug accumulation. Only 10% of the generated ligands inhibit OCT1, and 15% inhibit OCT2.

Nearly 85% of ligands show some degree of hepatotoxicity; this could be due to the inhibition of the bile salt export pump (BSEP), as all the generated ligands inhibit BSEP (Kenna et al., 2018). However, most (>90%) of the generated ligands are non-carcinogenic and non-mutagenic for Ames mutagenesis.

### 3.3. Drug likeness score prediction

**Figure 8:**
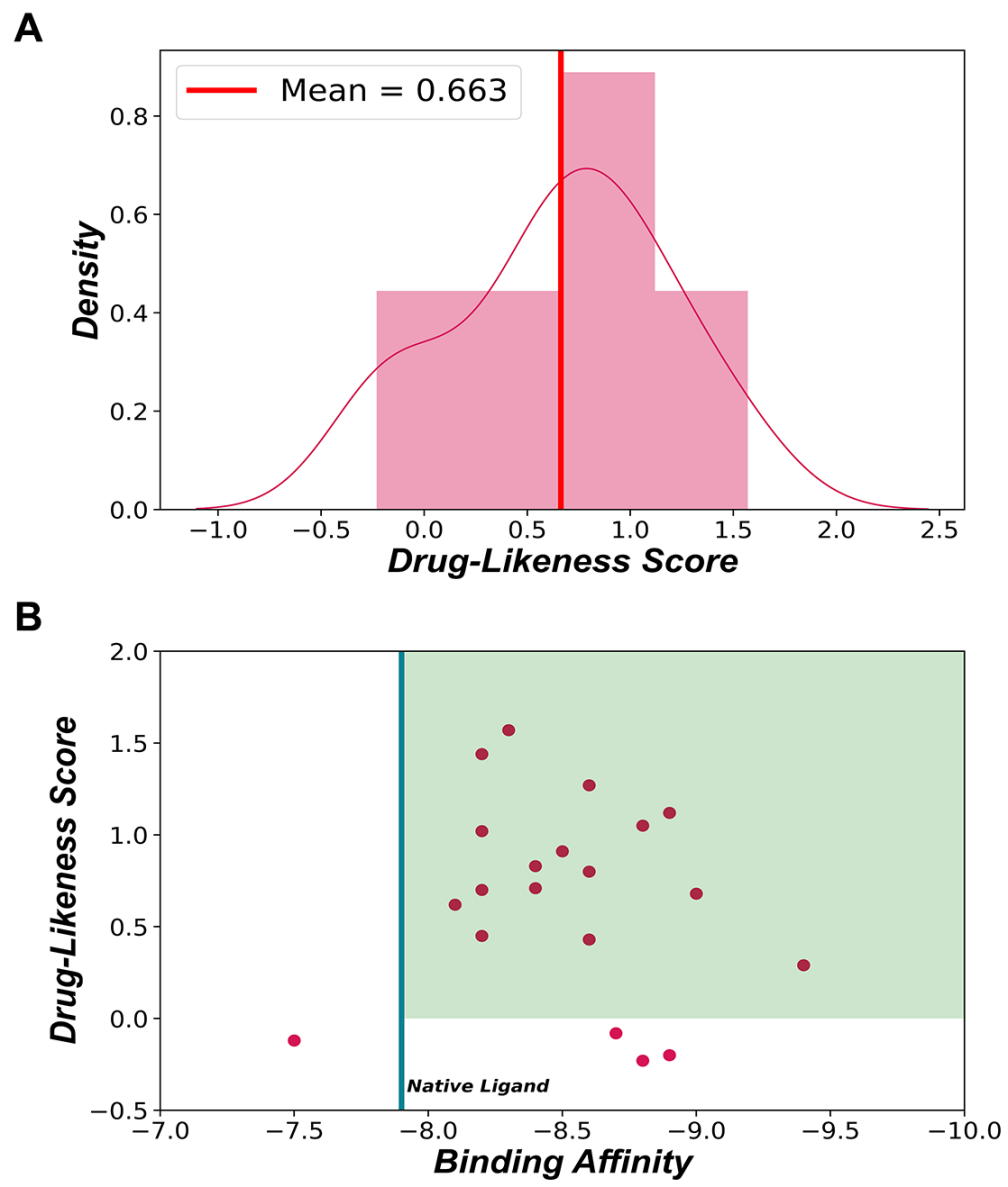
**(A)** Distribution of drug-likeness scores of generated ligands. The red line represents the average drug-likeness score. **(B)** Binding affinity vs. Drug-likeness score-generated ligands. The green region represents ligands showing good drug-likeness scores and having binding affinity greater than −7.9 kcal/mol.

The drug-likeness score of the generated ligands predicted by MolSoft are shown in Figure 8A. The average drug-likeness score was found to be 0.663 ± 0.118, and the scores of the generated ligands were found to be in the range with FDA reference drugs used by MolSoft. Drugs that have drug–likeness scores greater than 0 are considered to have drug-like properties (Prabhavathi et al., 2020). In Figure 8B, ligands that fall in the green region have a binding affinity greater than −7.9 kcal/mol and have a drug-likeness score greater than 0. 16 (80%) ligands fall under the green region, indicating that these ligands have a good potential to be developed as lead molecules.

### 3.4. Post docking analysis

**Figure 9:**
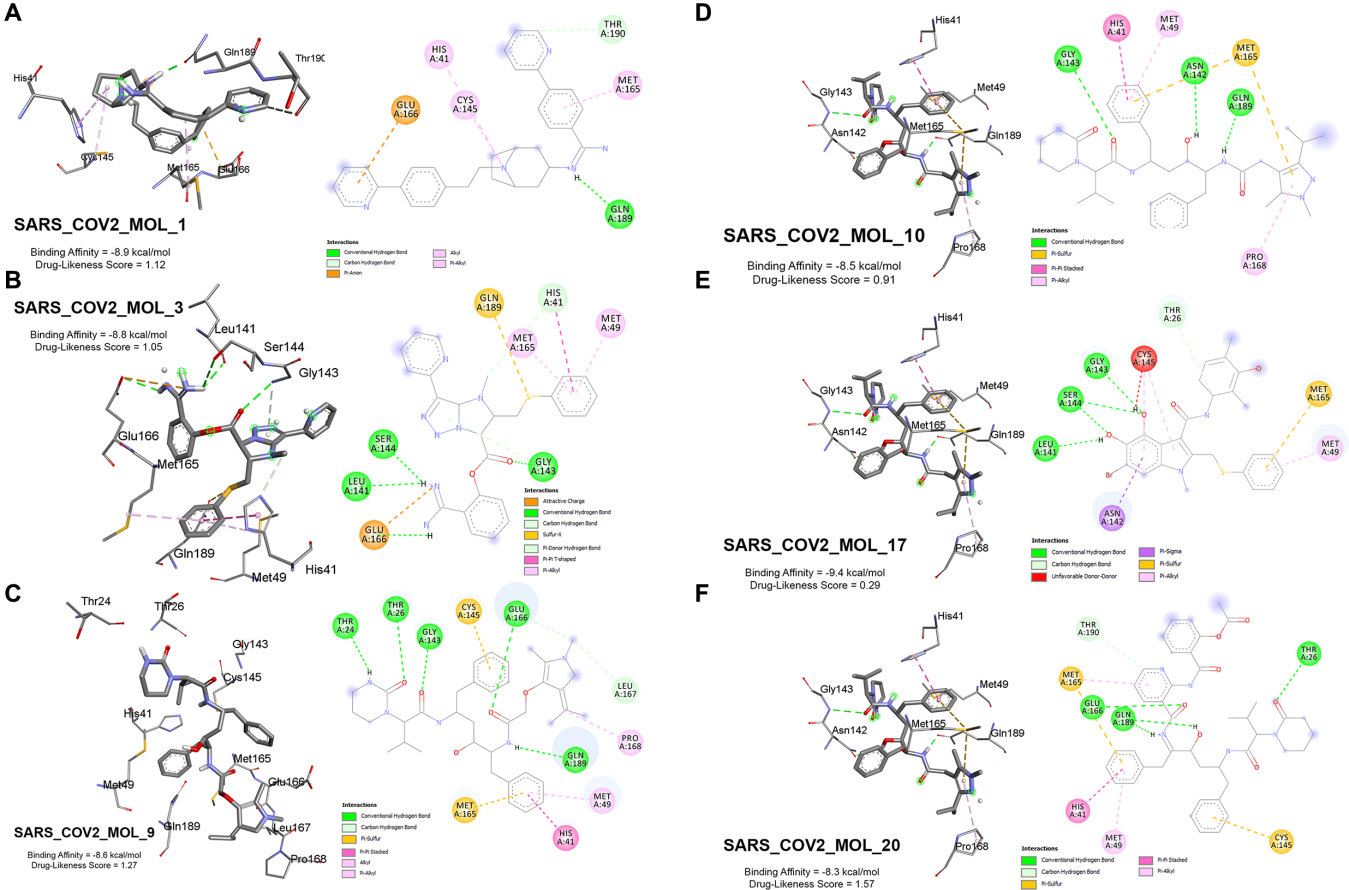
Docked 3D and 2D interaction with 3CL^pro^ (6 LU7, **A–F**).

From the 20 generated ligands, 6 were selected to study their interactions with 3CL^pro^ using Discovery Studio. Figures 9A–F, displays the interaction of the six ligands with the protease, and the amino acid residues interacting with the ligand have been annotated. Hydrogen bonds are an essential factor that determines the stability of the docked complex. Most of the ligands can be seen interacting with receptor residues Leu 141, Asn 142, Gly 143, and Ser 144 via a hydrogen bond. SARS_COV2_MOL_17 (Figure 9E) shows the highest binding affinity of −9.4 kcal/mol and a drug-likeness score of 0.29. The ligand showed hydrogen bond interaction with receptor residues at Leu 141, Gly 143, and Ser 144. However, there is an unfavorable donor-donor interaction at Cys 145. Unfavorable bonds greatly hinder the stability of the protein-ligand complex. SARS_COV_MOL_20 (Figure 9F) has the highest drug-likeness score of 1.57 and a binding affinity of −8.3 kcal/mol. This ligand can be seen interacting with hydrogen bonds at receptor residues Thr 26, Glu 166, and Gln 189. However, among the six ligands, SARS_COV2_MOL_1, 3, 9, and 10 (Figures 9A–D respectively) have shown high binding affinity with a good drug-likeness score and less than 1 violation of Lipinski’s Rule. SARS_COV2_MOL_9 has shown a high drug-likeness score of 1.27 and is also a good protease inhibitor (Protease inhibitor bioactivity score - 0.26 (refer Appendix-A)). It also exhibits a high number of hydrogen bond interactions at the receptor (Thr 24, Thr 26, Gly 143, Glu 166, and Gln 189) and interacts with a pi-pi stacked bond at His 41 and pi-alkyl bond at Pro 168, and Met 49. In conclusion, the generated ligands are seen to be forming stable complexes with 3CL^pro^ protease having high binding affinities.

### 3.5. MD simulations analysis

To further validate the structural stability of the generated ligands, MD simulations was performed on the docked complexes for 100 ns. The trajectories obtained after the simulations were analyzed to calculate RMSD, RMSF, hydrogen bonds, Rg, and SASA to assess the stability of the simulated systems.

### 3.6. Root-mean-square deviation

**Figure 10:**
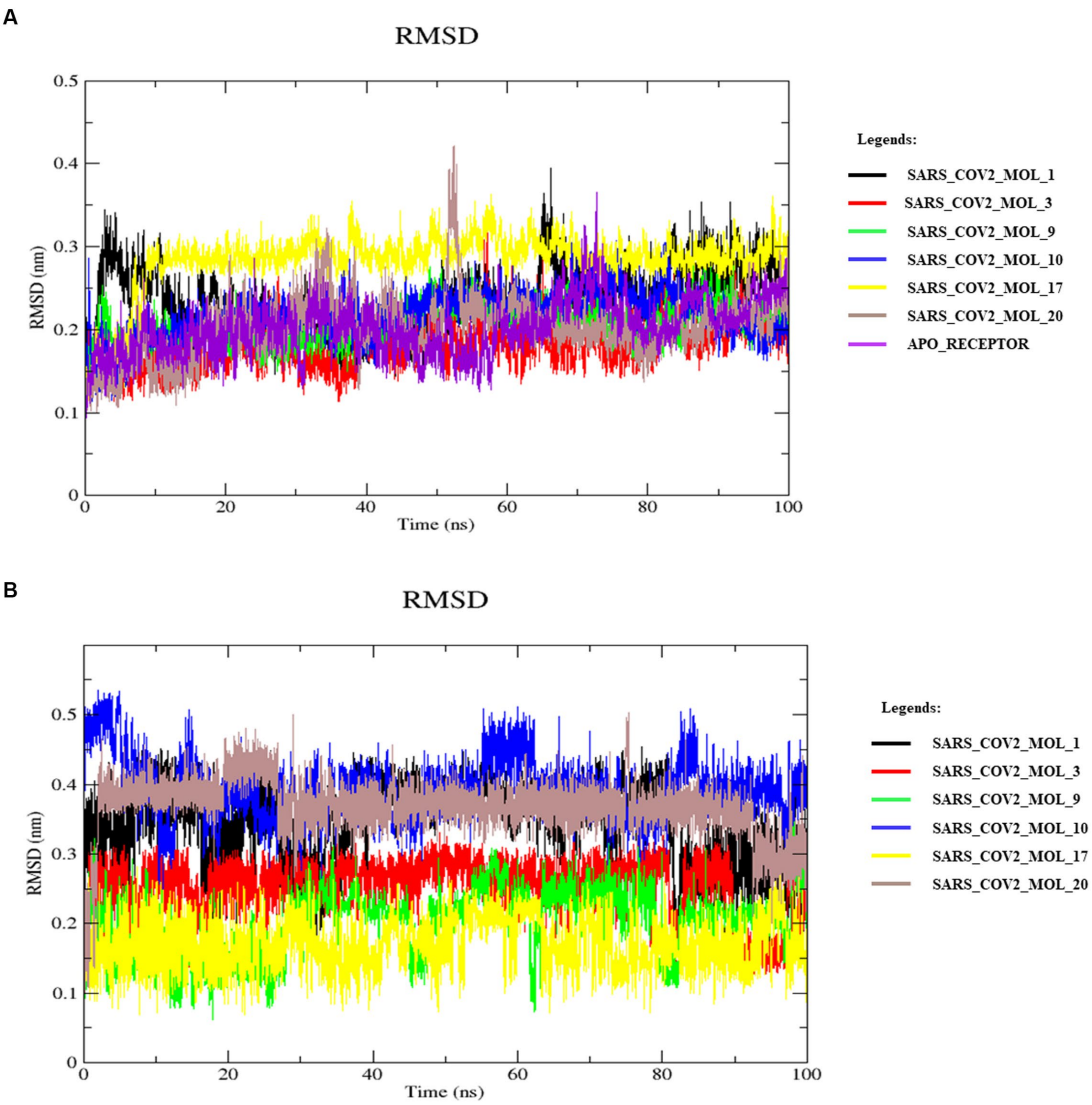
RMSDs of the receptor **(A)** backbone atoms and ligands **(B)** during MD simulation.

RMSD is commonly used to evaluate docked complex stability (Martínez, 2015; Sargsyan et al., 2017). It measures the difference between the initial position and the final conformation of the protein backbone. From Figure 10, the SARS_COV2_MOL_3 (red) complex showed the lowest average RMSD value, around 0.18 nm, among all six complexes. The average RMSD values of the other five complexes with SARS_COV2_MOL_1 (black), SARS_COV2_MOL_9 (green), SARS_COV2_MOL_10 (blue), SARS_COV2_MOL_17 (yellow), and SARS_COV2_MOL_20 (brown) was estimated to be ~0.2 nm same as that of the apoprotein. The RMSD values remained nearly constant over the 100 ns period, indicating that the protein-ligand complexes were structurally stable (Figure 9A). The low RMSD values suggest that the protein-ligand interactions were energetically favorable and contributed to the stability of the complexes. The SARS_COV2_MOL_20 and SARS_COV2_MOL_1 complexes showed slight deviations of ~0.4 and ~0.38 nm around 50 and 66 ns, respectively. These fluctuations were further confirmed by observing local changes at the residue level using the RMSF plot.

**Figure 11:**
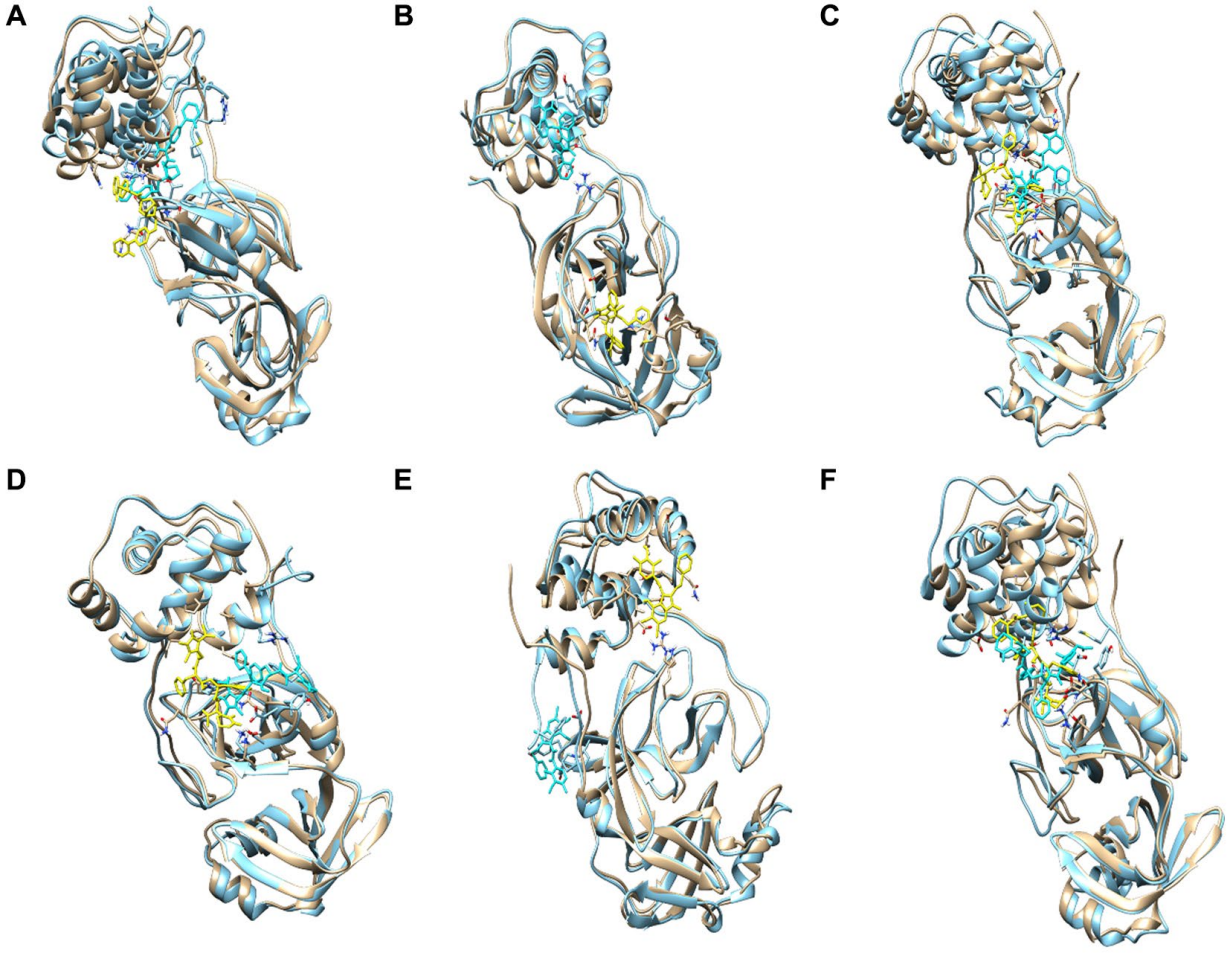
Superimposition of the post-MD complexes of **(A)** SARS_COV2_MOL_1, **(B)** SARS_COV2_MOL_3, **(C)** SARS_COV2_MOL_9, **(D)** SARS_COV2_MOL_10, **(E)** SARS_COV2_MOL_17, and **(F)** SARS_COV2_MOL_20, with initial docking pose of the respective complexes.

The ligand RMSD ranged between 0.16–0.39 nm as shown in Figure 9B. SARS_COV2_MOL_17 had the least average RMSD which suggest the stability of the protein-ligand system when bound with SARS_COV2_MOL_17. Rests of the ligands also showed no sharp deviation and were stable throughout the period of simulation. It indicates the stability of the complexes. Alignment of the post-MD complexes with their respective initial-docking poses corroborate the low deviations observed, as illustrated in Figure 11.

### 3.7. Root-mean-square fluctuation

**Figure 12:**
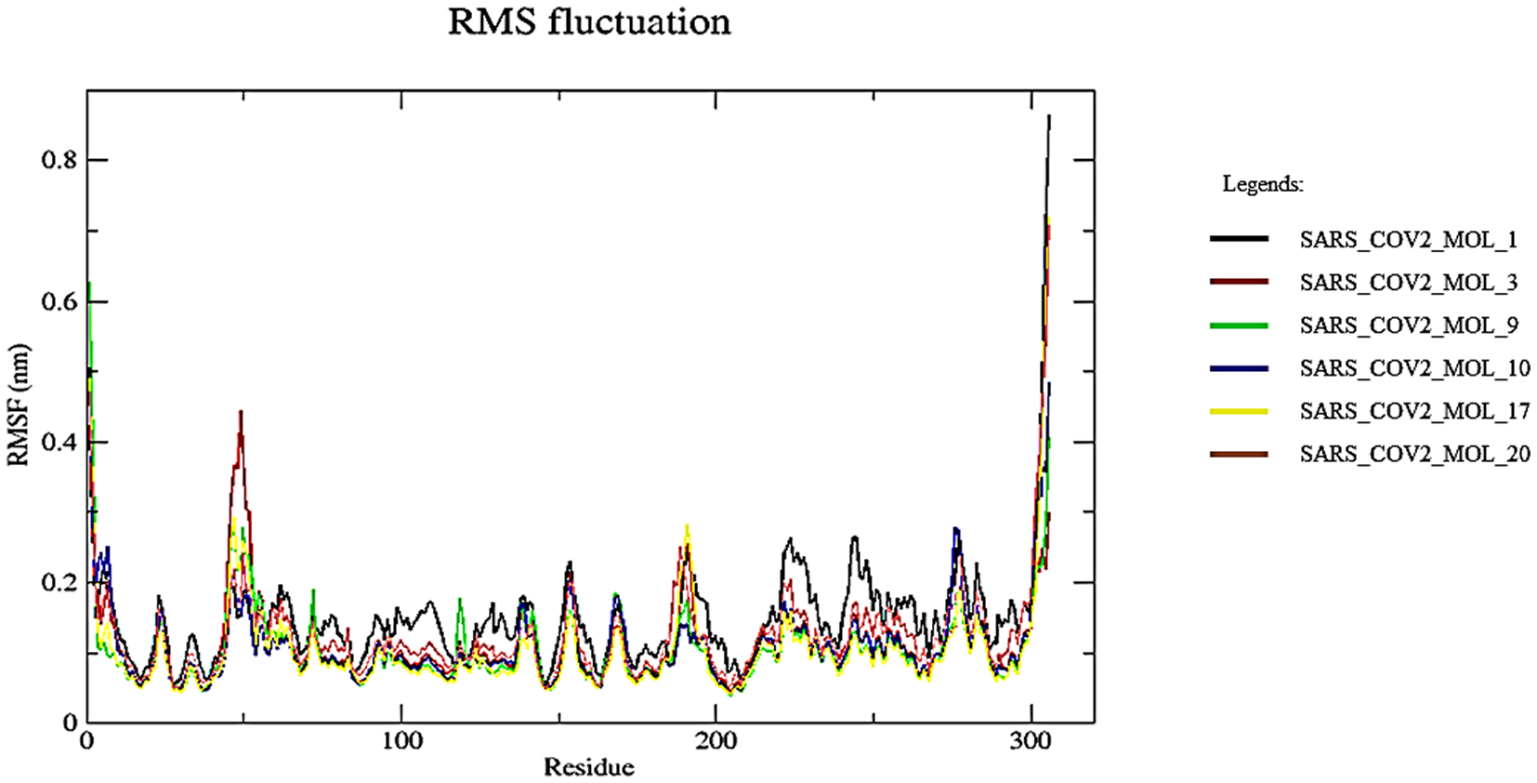
RMSF analysis of Cα during MD simulation.

The fluctuations in the protein can be determined by calculating RMSF, which measures the flexibility of each residue over time. The stability of the protein-ligand complexes can be inferred from the RMSF scores, with higher values indicating less stability and more flexibility. The RMSF of Cα atoms was calculated for all complexes, and the resulting average values for the six ligands were between 0.103 and 0.150 nm, as shown in Figure 12. The RMSF of the apoprotein was around 0.134 nm with no major fluctuation with respect to the protein-ligand complexes. However, the residues in the range of 46–50 showed slight fluctuation of average 0.37 nm. These low RMSF values suggest that the protein-ligand complexes are relatively stable and exhibit a moderate degree of flexibility. The close proximity of the complexes with the apoprotein also indicates about the stability of the complexes. This indicates that the ligands can bind to the protein without causing significant changes in its conformation. So, the predicted system appears to be stable (Farmer et al., 2017).

### 3.8. Radius of gyration and solvent accessible surface area analysis

**Figure 13:**
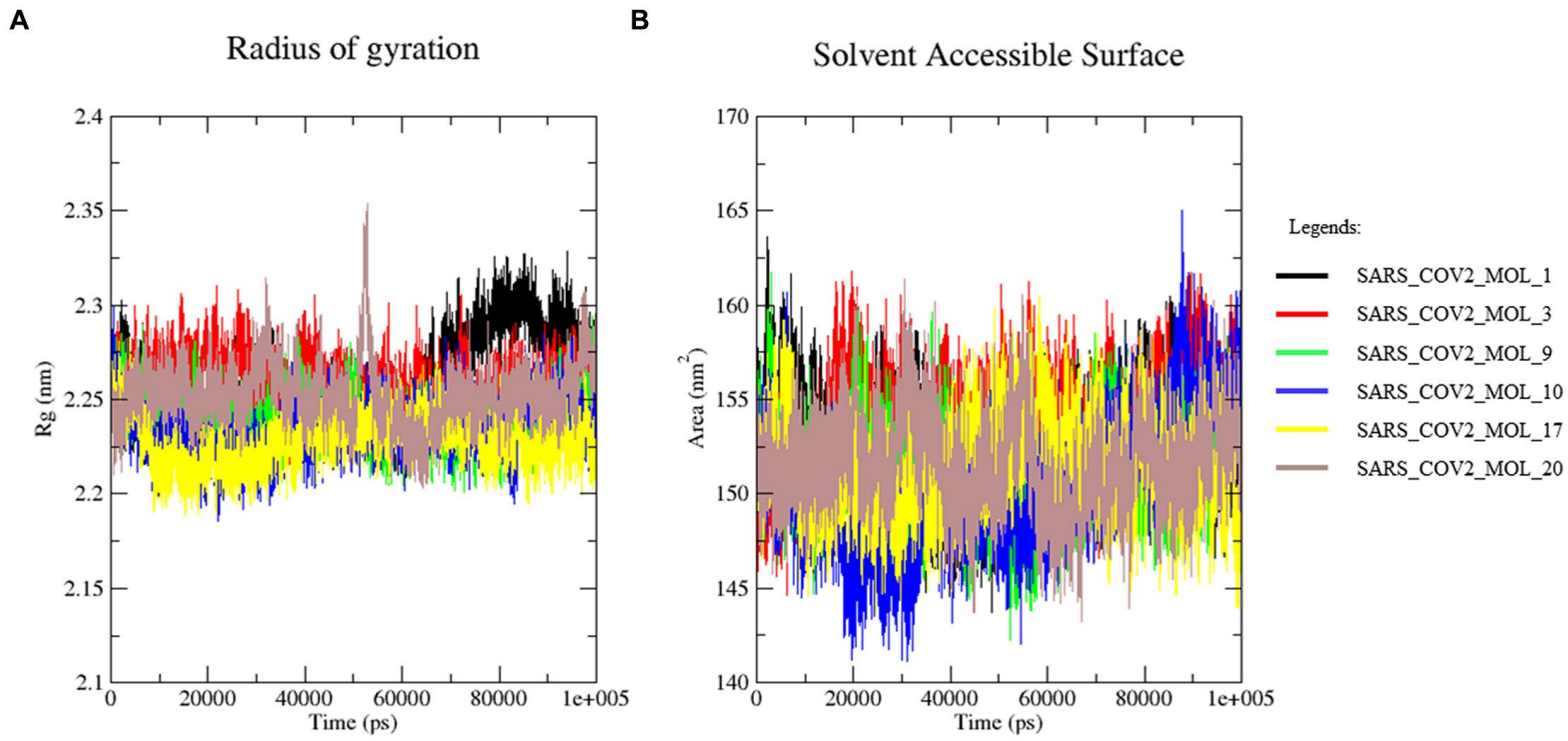
**(A)** Radius of gyration and **(B)** solvent accessible surface area.

The compactness and stability of protein structures can be measured using the Rg, which represents the mass-weighted root mean square distance of the atomic distribution from their mutual center of mass. The Rg values depict the inclusive dimensions of the protein and protein-ligand complexes and reflect their appropriate interactions. The protein-ligand complexes that displayed the least radius of gyration are considered to be more compact and stable. The Rg values of all the complexes were analyzed, and the solvent-accessible surface area (SASA) was also computed for all the proteins for 100 ns. The SASA is an important measure to determine the area of the receptor exposed to the solvents during the simulation. As shown in Figure 13A, all the systems exhibited similar Rg values, ranging from 2.2 to 2.6 nm throughout the simulation, indicating their stability. The estimated SASA values also displayed a similar pattern, varying between 150.59 and 153.93 nm^2^, as depicted in Figure 13B, with the highest value observed for the SARS_COV2_MOL_3 complex. These observations confirm the stability and compactness of all the protein-ligand complexes, as smaller deviations in average Rg and SASA values (Figure 13) Suggesting a stronger binding between the protein and ligand (Shaji, 2016).

### 3.9. Hydrogen bond analysis

**Figure 14:**
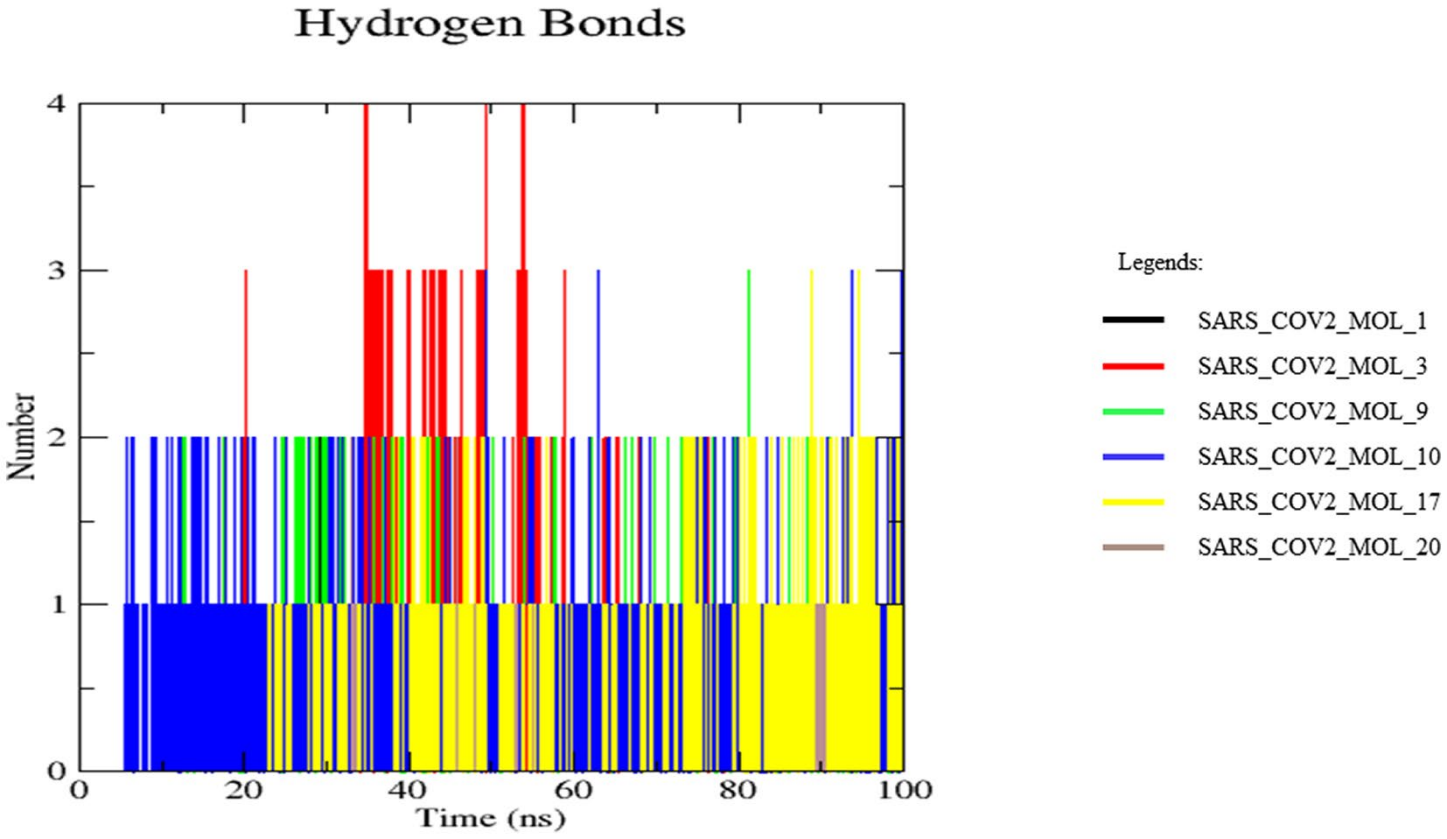
Hydrogen bond analysis of the docked complexes.

The stability and molecular recognition process of a protein-ligand complex is affected by the intermolecular hydrogen bonds (H-bonds) between interacting atom pairs. The number of H-bonds formed between the receptor protein and selected ligands was determined during the 100 ns MD simulations to ascertain the dynamic stability of each complex. The binding strength and specificity of the protein-ligand complex are determined by hydrogen bonds. Figure 14, represents the number of hydrogen bonds formed between the receptor protein and selected ligands throughout the MD simulation. The complex formed with SARS_COV2_MOL_3 (red) showed a higher number of hydrogen bonds, while the rest of the complexes showed a stable number of hydrogen bonds throughout the 100 ns simulation (Figure 12). The results further suggest the stability of the studied 3CL^pro^ inhibitors (Pereira et al., 2019; Zhu et al., 2022).

## 4. Discussion

Here, the properties of ligands produced by a deep neural network (RNN-LSTM) to inhibit 3CL^pro^ were assessed and compared to drugs currently undergoing clinical trials as a potential treatment for COVID-19. The findings demonstrate the RNN-LSTM’s ability to produce new ligands with strong binding affinities to 3CL^pro^, outperforming the native ligand and reference drugs in most cases. The selection of ligands was primarily based on their binding affinity to the target protease, which is a crucial parameter here. Similar methods for screening and selecting ligands were employed by Meng et al. (2011) and Hernández-Santoyo et al. (2013). *In-silico* docking studies with drugs such as Ritonavir, Lopinavir, Remdesivir, and Benzophenone derivatives have suggested the potential of these drugs to inhibit viral proteases (Bhardwaj et al., 2020; Li et al., 2020; Li and Kang, 2020; Rujuta et al., 2020). Polymerase inhibitors such as Sofosbuvir, Remdesivir, Tenofovir, Ribavirin, and Galidesivir have also been identified as promising inhibitors based on their binding energies (Ju et al., 2020a,b). The binding energy of inhibitors, including nucleotide analogs, is a crucial parameter for assessing their potential to inhibit viral replication (Udofia et al., 2021).

Of the 20 ligands chosen, most exhibit physicochemical properties that satisfy Lipinski’s Rule of 5, but some deviate from it in terms of molecular weight. Nonetheless, a molecular weight of 500 Da alone is not a reliable predictor of oral bioavailability and drug-likeness. Studies have shown that compounds having ten or fewer rotatable bonds and a total polar surface area (TPSA) of less than 140 have a higher probability of oral bioavailability and drug-likeness (Veber et al., 2002). And 10 of the generated ligands satisfy this criterion (Figure 3F). The drug-likeness potential was further reinforced by the results from Molinspiration and Molsoft. ADMET analysis revealed that the ligands have an acceptable ADME profile and showed very low toxicity. Leeson et al. (2021) discussed the ability of physicochemical descriptors commonly used to define “drug-likeness” and ligand efficiency measures to differentiate marketed drugs from compounds reported to bind to their efficacious target or targets. The study found that recent drugs approved in 2010–2020 had no overall differences in molecular weight, lipophilicity, hydrogen bonding, or polar surface area from the marketed compounds. However, drugs differed by higher potency, ligand efficiency (LE), lipophilic ligand efficiency (LLE), and lower carboaromaticity (Leeson et al., 2021). The ligands generated by the model can be further improved by repeating the fine-tuning process using a dataset of generated ligands with these desirable properties.

Molecular dynamic simulation studies at 100 ns revealed that the generated ligands formed stable complexes with 3CL^pro^. The root-mean-square deviation was 0.18 nm and ~0.2 nm, with a root-mean-square fluctuation ranging from 0.103 to 0.150 nm, a solvent-accessible surface area between 150.59 and 153.93 nm2, a radius of gyration ranging from 2.2 to 2.6 nm, and a stable number of hydrogen bonds. The results suggest that these ligands form strong and stable complexes with 3CL^pro^. As docking and simulation studies using *in silico* tools are well accepted for various purposes (Paital et al., 2015; Shaji, 2016; Farmer et al., 2017; Mishra et al., 2019; Pereira et al., 2019; Bulut et al., 2020; Hou et al., 2023; Sahoo et al., 2022; Zhu et al., 2022), the present study may be useful for targeting 3CL^pro^.

Overall, the generated ligands demonstrated comparable or even superior drug-like properties when compared to the drugs currently undergoing clinical trials, making them a promising candidate for further development in the treatment of COVID-19.

## 5. Conclusion

In the current study, a deep RNN was trained to produce novel ligands that could potentially inhibit the main viral protease of SARS-CoV-2, 3CL^pro^ (PDB: 6 LU7), and 20 novel ligands were identified. The study’s results unequivocally indicate that the novel ligands produced by the deep generative model have the potential to serve as effective anti-COVID drugs. Furthermore, the study adds to the growing body of evidence supporting the use of deep learning as a means of expediting drug discovery. However, further testing by *in vitro* and *in vivo* studies is necessary before considering them for human use.

## Data availability statement

The original contributions presented in the study are included in the article/Supplementary material, further inquiries can be directed to the corresponding authors.

## Author contributions

PP, BP, SVM, and DS: conceptualization, data curation, formal analysis, funding acquisition, investigation, methodology, project administration, resources, software, supervision, validation, visualization, roles/writing—original draft, and writing—review and editing. AH, SM, MS, SK and PD: concept, writing—original draft, and writing—review and editing. All authors contributed to the article and approved the submitted version.

## Funding

This work was supported by Schemes (number ECR/2016/001984 from Science Engineering Research Board, DST, Govt. of India and 1188/ST, Bhubaneswar, dated 01.03.17, ST- (Bio)-02/2017 from Department of Biotechnology, DST, Govt. of Odisha, India) to BP are acknowledged.

## Conflict of interest

The authors declare that the research was conducted in the absence of any commercial or financial relationships that could be construed as a potential conflict of interest.

## Publisher’s note

All claims expressed in this article are solely those of the authors and do not necessarily represent those of their affiliated organizations, or those of the publisher, the editors and the reviewers. Any product that may be evaluated in this article, or claim that may be made by its manufacturer, is not guaranteed or endorsed by the publisher.

## Footnotes

^1^ https://covid19.who.int/

^2^ https://www.tensorflow.org

^3^ https://keras.io

^4^ https://www.python.org

^5^ https://www.rdkit.org

^6^ https://www.ebi.ac.uk/chembl

^7^ https://pubchem.ncbi.nlm.nih.gov/#query=covid-19&tab=bioassay

^8^ https://www.rcsb.org/pdb/

^9^ http://www.molinspiration.com

^10^ http://molsoft.com/mprop

^11^ http://lmmd.ecust.edu.cn/admetsar2/

^12^ https://cgenff.umaryland.edu

## Abbreviations

3CLpro: 3 Chymotrypsin-like protease
ADMET: Absorption, distribution, metabolism, excretion and toxicity
BSEP: Bile salt export pump
HBA: Hydrogen bond acceptors
HBD: Hydrogen bond donors
MW: Molecular weight
NRB: Number of rotatable bonds
OCT: Organic cation transporters
RNN: Recurrent neural network
SDF: Structure data file format
SMILES: Sequence of the molecule in the simplified molecular-input line-entry system
TPSA: Total polar surface area
hERG: Human ether-a-go-go
