# Supplementary material for: Insilico generation of novel ligands for the inhibition of SARS-CoV-2 main protease (3CLpro) using deep learning
Source: Front Microbiol. 2023 Jun 23;14:1194794. doi: 10.3389/fmicb.2023.1194794 (PMC10338188; doi:10.3389/fmicb.2023.1194794)
Supplement: Supplementary file 1 [file Data_Sheet_1.docx]

**Structural details of 3CL pro**


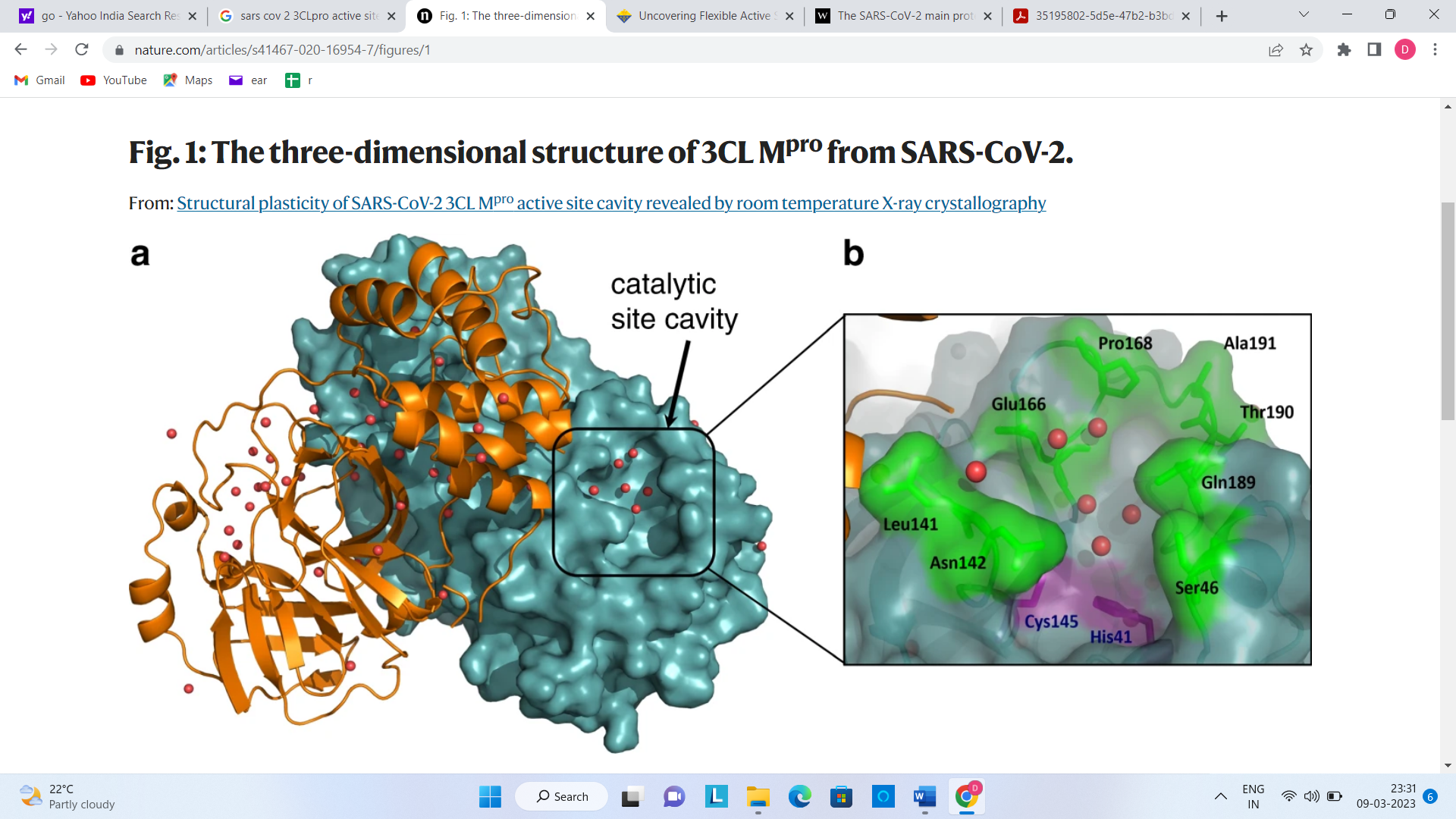


Reference: Zhu et al. 2022

**Table: Residues and recognition sequences of active site of 3CLpro**

| **Substrate binding pockets** | **Major residues in the active site of 3CL pro** | **Recognition sequences** |
| --- | --- | --- |
| S1 | L27, H41, Cys145 | Ala, Ser, Gly, Asn |
| S1 | H163, E166, E140, G143, S144 | Gln |
| S2 | H41, M49, M165, Val86, D187, R188, Q189 | Leu, Phe, Met, Val |
| S3 | E166 | Thr, Lys, Arg, Val, Leu |
| S4 | M165, L167, Q189, T190, A191 | Ala, Val, Pro, Thr |
| S5 | T190, A191, Q192 | Val, Ala, Glu, Phe, Gly, His, Arg, Ser, Thr, Tyr |

Source: Zhu J, Zhang H, Lin Q, Lyu J, Lu L, Chen H, Zhang X, Zhang Y, Chen K. 2022. Progress on SARS-CoV-2 3CLpro Inhibitors: Inspiration from SARS-CoV 3CLpro Peptidomimetics and Small-Molecule Anti-Inflammatory Compounds. Drug Des Devel Ther. 16:1067-1082. doi: 10.2147/DDDT.S359009.

Appendix A: Reference Drugs and Generated Ligands

Reference Drugs

| Binding Affinity | Enzyme Inhibitor | GPCR Ligand | Identifier | Ion Channel Modulator | Kinase Inhibitor | MW | Name | Nuclear Receptor Inhibitor | Protease Inhibitor | Smiles | TPSA | miLogP | nOHNH | nON | natom | nviolations | Ames mutagenesis | Acute Oral Toxicity (c) | Androgen receptor binding | Aromatase binding | Blood Brain Barrier | BRCP inhibitior | BSEP inhibitior | Caco-2 | Carcinogenicity (binary) | Carcinogenicity (trinary) | CYP1A2 inhibition | CYP2C19 inhibition | CYP2C9 inhibition | CYP2C9 substrate | CYP2D6 inhibition | CYP2D6 substrate | CYP3A4 inhibition | CYP3A4 substrate | CYP inhibitory promiscuity | Eye corrosion | Eye irritation | Estrogen receptor binding | Glucocorticoid receptor binding | Hepatotoxicity | Human either-a-go-go inhibition | Human Intestinal Absorption | Human oral bioavailability | MATE1 inhibitior | micronuclear | Acute Oral Toxicity | OATP1B1 inhibitior | OATP1B3 inhibitior | OATP2B1 inhibitior | OCT1 inhibitior | OCT2 inhibitior | P-glycoprotein inhibitior | P-glycoprotein substrate | PPAR gamma | Plasma protein binding | Subcellular localzation | Thyroid receptor binding | UGT catelyzed | Water solubility |
| --- | --- | --- | --- | --- | --- | --- | --- | --- | --- | --- | --- | --- | --- | --- | --- | --- | --- | --- | --- | --- | --- | --- | --- | --- | --- | --- | --- | --- | --- | --- | --- | --- | --- | --- | --- | --- | --- | --- | --- | --- | --- | --- | --- | --- | --- | --- | --- | --- | --- | --- | --- | --- | --- | --- | --- | --- | --- | --- | --- |
| -7.9 | 0.38 | 0.27 | Reference Drugs | -0.35 | 0.2 | 602.59 | Remdesivir | -0.48 | 0.49 | CCC(CC)COC(=O)C(C)NP(=O)(OCC1C(C(C(O1)(C#N)C2=CC=C3N2N=CN=C3N)O)O)OC4=CC=CC=C4 | 203.57 | 2.82 | 5 | 14 | 42 | 2 | - | III | + | + | + | - | + | - | - | Non-required | - | - | - | - | - | - | - | + | - | - | - | + | + | + | - | + | - | - | + | 3.427931309 | + | + | - | - | - | + | + | + | 1.182161927 | Lysosomes | + | - | -3.473555922 |
| -5.9 | 0.11 | 0.32 | Reference Drugs | 0.32 | 0.38 | 319.88 | Chlorquine | -0.19 | 0.05 | CCN(CC)CCCC(C)NC1=C2C=CC(=CC2=NC=C1)Cl | 28.16 | 5 | 1 | 3 | 22 | 1 | + | II | - | + | + | - | + | + | - | Non-required | - | - | - | - | - | + | - | + | - | - | - | - | - | + | + | + | + | - | + | 2.683706999 | + | + | - | + | - | - | + | + | 0.862241209 | Lysosomes | + | - | -4.348487817 |
| -7.3 | -0.74 | -0.33 | Reference Drugs | -1.41 | -1.02 | 720.96 | Ritonavir | -1.41 | 0.35 | CC(C)C1=NC(=CS1)CN(C)C(=O)NC(C(C)C)C(=O)NC(CC2=CC=CC=C2)CC(C(CC3=CC=CC=C3)NC(=O)OCC4=CN=CS4)O | 145.78 | 7.51 | 4 | 11 | 50 | 3 | - | III | + | + | - | - | + | - | - | Non-required | - | + | - | + | - | - | + | + | + | - | - | + | + | + | + | + | - | - | + | 2.281080484 | + | + | + | - | - | + | + | + | 1.112688661 | Lysosomes | + | - | -3.224976948 |
| -5 | 0.14 | 0.42 | Reference Drugs | 0.09 | 0.08 | 307.48 | FIngolimod | 0.02 | 0.29 | CCCCCCCCC1=CC=C(C=C1)CCC(CO)(CO)N | 66.48 | 4.72 | 4 | 3 | 22 | 0 | - | III | + | + | + | - | - | + | - | Non-required | + | - | - | - | + | + | - | - | - | - | - | + | - | - | - | + | - | - | - | 2.300637245 | + | + | - | - | - | - | + | + | 1.181407452 | Lysosomes | + | - | -2.009190588 |
| -6.4 | 1.15 | 0.78 | Reference Drugs | 0.03 | 0.36 | 259.22 | N(4)-Hydroxycytidine | -1.17 | 0.12 | C1=CN(C(=O)N=C1NO)C2C(C(C(O2)CO)O)O | 137.07 | -1.87 | 5 | 9 | 18 | 0 | - | III | + | + | + | - | - | - | - | Non-required | - | - | - | - | - | - | - | - | - | - | - | - | + | + | - | + | - | - | + | 2.080636025 | + | + | - | - | - | - | - | + | 0.456638277 | Mitochondria | + | - | -2.062430114 |
| -5 | -0.33 | -0.62 | Reference Drugs | -0.44 | -0.31 | 157.1 | Favipiravir | -1.5 | -0.91 | C1=C(N=C(C(=O)N1)C(=O)N)F | 88.95 | -0.98 | 3 | 5 | 11 | 0 | - | III | - | - | + | - | - | - | - | Non-required | - | - | - | - | - | - | - | - | - | - | - | - | - | + | - | + | + | - | + | 1.780337572 | + | + | - | - | - | - | - | - | 0.38049978 | Mitochondria | - | - | -1.651125689 |
| -7.6 | -0.37 | 0.04 | Reference Drugs | -0.78 | -0.55 | 628.81 | Lopinavir | -0.66 | 0.42 | CC1=C(C(=CC=C1)C)OCC(=O)NC(CC2=CC=CC=C2)C(CC(CC3=CC=CC=C3)NC(=O)C(C(C)C)N4CCCNC4=O)O | 119.99 | 5.69 | 4 | 9 | 46 | 2 | - | III | + | - | + | + | + | + | - | Non-required | - | - | - | - | - | - | - | + | - | - | - | + | + | + | + | + | - | - | + | 2.994427919 | + | + | + | - | - | + | + | + | 1.156630874 | Mitochondria | + | - | -3.414387961 |
| -7.9 | 0.31 | 0.35 | Reference Drugs | -0.21 | -0.24 | 547.67 | Darunavir | -0.26 | 1.15 | CC(C)CN(CC(C(CC1=CC=CC=C1)NC(=O)OC2COC3C2CCO3)O)S(=O)(=O)C4=CC=C(C=C4)N | 140.43 | 4.32 | 4 | 10 | 38 | 1 | - | III | + | - | + | - | + | + | - | Non-required | - | - | - | + | - | - | - | + | - | - | - | + | + | - | - | + | - | - | + | 4.535059929 | + | + | - | - | - | + | + | + | 1.034757853 | Lysosomes | - | + | -3.535724545 |
| -6.1 | 1.39 | 0.58 | Reference Drugs | 0.28 | 0.58 | 265.27 | Galidesivir | -0.74 | 0.09 | C1=C(C2=C(N1)C(=NC=N2)N)C3C(C(C(N3)CO)O)O | 140.31 | -2.24 | 7 | 8 | 19 | 1 | - | III | + | + | + | - | - | - | - | Non-required | - | - | - | - | - | - | - | - | - | - | - | - | - | + | - | + | + | - | + | 2.598165274 | + | + | - | - | - | - | - | + | 0.616459191 | Nucleus | + | + | -1.789851329 |
| -6.2 | 0.15 | 0.35 | Reference Drugs | 0.3 | 0.44 | 335.88 | Hydroxychloroquine | -0.12 | 0.12 | CCN(CCCC(C)NC1=C2C=CC(=CC2=NC=C1)Cl)CCO | 48.38 | 4 | 2 | 4 | 23 | 0 | + | III | - | + | + | - | + | + | - | Non-required | - | - | - | - | - | + | - | + | - | - | - | - | - | - | + | + | + | - | + | 2.664962053 | + | + | - | + | - | - | + | + | 0.756124914 | Lysosomes | + | + | -3.565742248 |
| -7.5 | 0.24 | 0.49 | Reference Drugs | 0.1 | 0.91 | 306.37 | Ruxolitinib | -0.67 | -0.13 | C1CCC(C1)C(CC#N)N2C=C(C=N2)C3=C4C=CNC4=NC=N3 | 83.19 | 1.83 | 1 | 6 | 23 | 0 | - | III | + | + | + | - | + | - | - | Non-required | + | - | - | - | - | - | - | + | + | - | - | + | - | - | + | + | + | - | + | 1.959711194 | + | + | - | - | + | - | + | + | 0.85203129 | Mitochondria | + | - | -2.168350321 |
| -7.6 | 0.11 | 0.27 | Reference Drugs | -0.12 | 0.62 | 371.43 | Baricitinib | -0.76 | -0.03 | CCS(=O)(=O)N1CC(C1)(CC#N)N2C=C(C=N2)C3=C4C=CNC4=NC=N3 | 120.57 | -0.24 | 1 | 9 | 26 | 0 | - | III | + | + | + | - | + | - | - | Non-required | - | - | - | - | - | - | - | + | - | - | - | + | + | + | - | + | + | - | + | 2.89871335 | + | + | - | - | - | - | + | + | 0.877454698 | Lysosomes | + | - | -3.160187175 |
| -7 | -0.07 | -0.19 | Reference Drugs | -0.44 | -0.39 | 477.42 | Arbidol | -0.34 | -0.46 | CCOC(=O)C1=C(N(C2=CC(=C(C(=C21)CN(C)C)O)Br)C)CSC3=CC=CC=C3 | 54.7 | 4.86 | 1 | 5 | 29 | 0 | + | III | + | + | + | - | + | + | - | Non-required | + | + | + | - | - | - | - | + | + | - | - | + | + | + | - | + | - | - | + | 2.752530575 | + | + | - | + | - | + | - | + | 1.085685968 | Lysosomes | + | + | -4.367824619 |
| -6.4 | -0.79 | -1.71 | Reference Drugs | -0.54 | -0.97 | 228.19 | Triazavirin | -1.99 | -1.37 | CSC1=NC2=NN=C(C(=O)N2N1)[N+](=O)[O-] | 121.78 | -0.03 | 1 | 9 | 15 | 0 | + | III | - | + | + | - | - | - | - | Non-required | - | - | - | - | - | - | - | - | - | - | - | + | - | + | - | + | + | - | + | 1.709705591 | + | + | - | - | - | - | - | - | 0.630037963 | Mitochondria | + | - | -2.690848311 |
| -8.2 | -3.38 | -3.08 | Reference Drugs | -3.62 | -3.53 | 882.03 | Elbasir | -3.65 | -2.54 | CC(C)C(C(=O)N1CCCC1C2=NC=C(N2)C3=CC4=C(C=C3)N5C(OC6=C(C5=C4)C=CC(=C6)C7=CN=C(N7)C8CCCN8C(=O)C(C(C)C)NC(=O)OC)C9=CC=CC=C9)NC(=O)OC | 188.82 | 8.85 | 4 | 16 | 65 | 3 | - | III | + | + | + | - | + | - | - | Non-required | + | + | - | - | - | - | + | + | + | - | - | + | + | + | + | + | - | - | + | 3.115997076 | + | + | + | - | - | + | + | + | 1.286148191 | Mitochondria | + | - | -3.510106103 |
| -6.1 | 0.71 | 0.31 | Reference Drugs | 0.21 | -0.21 | 244.21 | Ribavirin | -1.46 | -0.2 | C1=NC(=NN1C2C(C(C(O2)CO)O)O)C(=O)N | 143.73 | -2.77 | 5 | 9 | 17 | 0 | - | III | - | + | + | - | - | - | - | Non-required | - | - | - | - | - | - | - | - | - | - | - | - | - | + | - | + | - | - | + | 2.004169941 | + | + | - | - | - | - | - | + | 0.113588288 | Mitochondria | - | + | -1.172809894 |
| -7.4 | -0.08 | -0.1 | Reference Drugs | -0.15 | -0.32 | 398.42 | Camostat | -0.2 | 0.07 | CN(C)C(=O)COC(=O)CC1=CC=C(C=C1)OC(=O)C2=CC=C(C=C2)N=C(N)N | 137.33 | 1.56 | 4 | 9 | 29 | 0 | - | III | + | - | + | - | + | - | - | Non-required | - | - | - | + | - | - | - | + | - | - | - | + | + | + | + | + | + | + | + | 2.242214441 | + | + | - | - | - | + | - | + | 0.931325257 | Mitochondria | + | - | -3.520824349 |
| -5.2 | -2.83 | -2.99 | Reference Drugs | -3.5 | -0.36 | 914.19 | Sirolimus | -3.45 | -2.48 | CC1CCC2CC(C(=CC=CC=CC(CC(C(=O)C(C(C(=CC(C(=O)CC(OC(=O)C3CCCCN3C(=O)C(=O)C1(O2)O)C(C)CC4CCC(C(C4)OC)O)C)C)O)OC)C)C)C)OC | 195.45 | 4.92 | 3 | 14 | 65 | 2 | - | III | + | + | - | - | + | - | - | Non-required | - | - | - | - | - | - | - | + | - | - | - | + | + | + | + | + | - | - | + | 3.657267332 | + | + | - | - | - | + | + | + | 1.08860147 | Mitochondria | + | - | -2.900086206 |
| -8.6 | 0.19 | 0.28 | Reference Drugs | 0.14 | -0.03 | 347.28 | Nafamostat | -0.16 | 0.57 | C1=CC(=CC=C1C(=O)OC2=CC3=C(C=C2)C=C(C=C3)C(=N)N)N=C(N)N | 140.59 | 2.16 | 7 | 7 | 26 | 1 | - | III | + | + | + | - | + | - | - | Warning | + | - | - | - | - | - | - | - | - | - | - | + | + | + | - | + | - | + | + | 2.329654455 | + | + | - | - | - | - | - | + | 0.739231646 | Mitochondria | + | - | -4.073750382 |
| -6.9 | 0.71 | 0.07 | Reference Drugs | -0.28 | -0.72 | 374.48 | Methyprednisone | 1.22 | 0.3 | CC1CC2C3CCC(C3(CC(C2C4(C1=CC(=O)C=C4)C)O)C)(C(=O)CO)O | 94.83 | 2.07 | 3 | 5 | 27 | 0 | - | III | + | + | - | - | + | - | - | Non-required | - | - | - | - | - | - | - | + | - | - | - | + | + | - | + | + | + | - | - | 2.806599379 | + | + | - | - | + | - | - | - | 0.927034199 | Mitochondria | + | + | -3.243689196 |
|  |  |  |  |  |  |  |  |  |  |  |  |  |  |  |  |  |  |  |  |  |  |  |  |  |  |  |  |  |  |  |  |  |  |  |  |  |  |  |  |  |  |  |  |  |  |  |  |  |  |  |  |  |  |  |  |  |  |  |  |

Generated ligands

| BBB score | Binding Affinity | Drug-Likeness Score | Enzyme Inhibitor | GPCR Ligand | Identifier | Ion Channel Modulator | Kinase Inhibitor | MW | Name | Nuclear Receptor Inhibitor | Protease Inhibitor | Smiles | NRB | TPSA | miLogP | nOHNH | nON | natom | nviolations | Ames mutagenesis | Acute Oral Toxicity (c) | Androgen receptor binding | Aromatase binding | Blood Brain Barrier | BRCP inhibitior | BSEP inhibitior | Caco-2 | Carcinogenicity (binary) | Carcinogenicity (trinary) | CYP1A2 inhibition | CYP2C19 inhibition | CYP2C9 inhibition | CYP2C9 substrate | CYP2D6 inhibition | CYP2D6 substrate | CYP3A4 inhibition | CYP3A4 substrate | CYP inhibitory promiscuity | Eye corrosion | Eye irritation | Estrogen receptor binding | Glucocorticoid receptor binding | Hepatotoxicity | Human either-a-go-go inhibition | Human Intestinal Absorption | Human oral bioavailability | MATE1 inhibitior | micronuclear | Acute Oral Toxicity | OATP1B1 inhibitior | OATP1B3 inhibitior | OATP2B1 inhibitior | OCT1 inhibitior | OCT2 inhibitior | P-glycoprotein inhibitior | P-glycoprotein substrate | PPAR gamma | Plasma protein binding | Subcellular localzation | Thyroid receptor binding | UGT catelyzed | Water solubility |
| --- | --- | --- | --- | --- | --- | --- | --- | --- | --- | --- | --- | --- | --- | --- | --- | --- | --- | --- | --- | --- | --- | --- | --- | --- | --- | --- | --- | --- | --- | --- | --- | --- | --- | --- | --- | --- | --- | --- | --- | --- | --- | --- | --- | --- | --- | --- | --- | --- | --- | --- | --- | --- | --- | --- | --- | --- | --- | --- | --- | --- | --- | --- |
| 3.77 | -8.9 | 1.12 | 0.16 | 0.47 | Generated Ligands | 0.3 | 0.31 | 487.65 | SC_1 | -0.06 | 0.41 | N=C(NC1CC2CCC(C1)N2CCc1ccc(-c2ccccn2)cc1)c1ccc(-c2ccccn2)cc1 | 8 | 64.9 | 4.6 | 2 | 5 | 37 | 0 | - | III | + | + | + | - | + | - | - | Non-required | - | - | - | - | + | - | - | + | + | - | - | + | + | - | + | + | - | - | + | 2.508381 | + | + | - | - | + | + | + | + | 0.91476 | Nucleus | + | - | -3.03195 |
| 1.34 | -8.9 | -0.2 | 0.05 | 0.17 | Generated Ligands | -0.01 | -0.36 | 487.55 | SC_2 | -0.06 | 0.51 | O=C(NC(=O)C(Cc1ccccc1)NC(=O)C1CCC2CCC(C1)C2C(=O)O)OCC1NCCO1 | 9 | 14306 | 1.8 | 4 | 10 | 35 | 0 | - | III | + | + | + | - | + | - | - | Non-required | - | - | - | + | - | - | - | + | - | - | - | + | + | + | - | + | - | - | + | 3.569991 | + | + | - | - | - | + | + | + | 0.813191 | Mitochondria | - | - | -2.46541 |
| 1.69 | -8.8 | 1.05 | 0.09 | 0.22 | Generated Ligands | -0.03 | -0.03 | 483.56 | SC_3 | -0.23 | 0.15 | Cn1c(CSc2ccccc2)c(C(=O)Oc2ccccc2C(=N)N)n2nnc(-c3ccccn3)c12 | 8 | 124.2 | 2.92 | 3 | 9 | 35 | 0 | - | III | + | + | + | - | + | - | - | Non-required | - | - | - | - | - | - | + | + | + | - | - | + | + | + | + | + | - | - | + | 2.340125 | + | + | - | - | - | + | + | + | 0.880234 | Mitochondria | + | - | -3.2716 |
| 2.15 | -8.8 | -0.23 | 0.13 | 0.24 | Generated Ligands | -0.04 | -0.29 | 470.57 | SC_4 | -0.12 | 0.41 | O=C(CC1NCCN1)NC(=O)C(Cc1ccccc1)NC(=O)C1CCC2CCC(C1)C2C(=O)O | 8 | 136.62 | 0.42 | 5 | 9 | 34 | 0 | - | III | + | + | + | - | + | - | - | Non-required | - | - | - | - | - | - | - | + | - | - | - | + | - | + | + | + | - | - | + | 3.877717 | + | + | - | - | - | + | + | + | 0.759677 | Mitochondria | - | - | -2.64617 |
| 2.03 | -8.7 | -0.08 | 0.18 | 0.29 | Generated Ligands | -0.01 | -0.17 | 461.52 | SC_5 | 0.12 | 0.35 | Cc1ccc(CC(=O)NC(Cc2ccccn2)C(=O)OC(Cc2ccccn2)C(=O)O)c(C)c1 | 11 | 118.49 | 2.4 | 2 | 8 | 34 | 0 | - | III | + | - | + | - | + | - | - | Non-required | - | - | - | + | - | - | - | + | - | - | - | + | - | + | + | - | + | - | + | 1.908764 | + | + | - | - | - | + | - | + | 1.080161 | Mitochondria | - | - | -2.96871 |
| 1.94 | -8.6 | 0.43 | 0.23 | 0.35 | Generated Ligands | 0.1 | -0.02 | 475.55 | SC_6 | -0.08 | 0.43 | Cc1cc(C(C)C)ncc1C(=O)NC(Cc1ccccn1)C(=O)NC(Cc1ccccn1)C(=O)O | 10 | 134.17 | -0.1 | 3 | 9 | 35 | 0 | - | III | + | - | + | - | + | - | - | Non-required | - | - | - | - | - | - | - | + | - | - | - | + | + | + | + | + | - | - | + | 1.66342 | + | + | - | - | - | + | - | + | 0.917077 | Mitochondria | + | - | -2.98499 |
| 2.45 | -8.1 | 0.62 | 0.08 | 0.03 | Generated Ligands | -0.18 | -0.16 | 497.58 | SC_7 | -0.12 | -0.07 | CC(=O)Oc1ccccc1CC(=O)c1c(CSc2ccccc2)n(C)c2c(-c3ccccn3)nnn12 | 9 | 91.4 | 3.89 | 0 | 8 | 36 | 0 | - | III | - | + | + | - | + | - | - | Non-required | - | + | - | - | - | - | + | + | + | - | - | + | + | + | + | + | - | - | + | 2.394265 | + | + | - | - | - | + | + | + | 1.05953 | Mitochondria | + | - | -3.37925 |
| 2.43 | -7.5 | -0.12 | 0.14 | 0.22 | Generated Ligands | 0.02 | -0.38 | 426.56 | SC_8 | 0.09 | 0.37 | C=C(CC)CNC(=O)C(Cc1ccccc1)NC(=O)C1CCC2CCC(C1)C2C(=O)O | 9 | 95.5 | 3.58 | 3 | 6 | 31 | 0 | - | III | + | - | + | - | + | - | - | Non-required | - | - | - | + | - | - | - | + | - | - | - | + | + | + | + | + | + | - | + | 3.136706 | + | + | - | - | - | + | + | + | 0.781721 | Mitochondria | - | - | -3.68883 |
| 1.26 | -8.6 | 1.27 | -0.53 | -0.12 | Generated Ligands | -1.06 | -0.78 | 659.87 | SC_9 | -0.85 | 0.26 | Cc1c(OCC(=O)NC(Cc2ccccc2)C(O)CC(Cc2ccccc2)NC(=O)C(C(C)C)N2CCCNC2=O)c(C(C)C)cn1C | 16 | 124.93 | 4.99 | 4 | 10 | 48 | 1 | - | III | + | - | + | + | + | - | - | Non-required | - | - | - | + | - | - | - | + | - | - | - | + | + | + | + | + | - | - | + | 3.293822 | + | + | + | - | - | + | + | + | 0.983814 | Mitochondria | + | - | -2.80686 |
| 1.2 | -8.5 | 0.91 | -0.44 | -0.04 | Generated Ligands | -0.95 | -0.68 | 644.86 | SC_10 | -0.86 | 0.28 | Cc1c(CC(=O)NC(Cc2ccccc2)C(O)CC(Cc2ccccc2)NC(=O)C(C(C)C)N2CCCNC2=O)c(C(C)C)nn1C | 15 | 128.59 | 4.89 | 4 | 10 | 47 | 1 | - | III | + | + | + | + | + | - | - | Non-required | - | - | - | - | - | - | - | + | - | - | - | + | + | + | + | + | - | - | + | 3.31244 | + | + | + | - | - | + | + | + | 1.003415 | Mitochondria | + | - | -2.81461 |
| 3.92 | -8.4 | 0.83 | 0.11 | 0.27 | Generated Ligands | 0.18 | 0.13 | 489.62 | SC_11 | 0.04 | 0.16 | O=C(OC1CC2CCC(C1)N2CCc1ccc(-c2ccccn2)cc1)c1ccc(-c2ccccn2)cc1 | 8 | 55.33 | 6.01 | 0 | 5 | 37 | 1 | - | III | + | - | + | - | + | - | - | Non-required | - | - | + | - | - | + | - | + | + | - | - | + | - | - | + | + | - | - | + | 2.288139 | + | + | - | + | + | + | + | + | 0.963845 | Mitochondria | - | - | -3.26662 |
| 4.27 | -8.4 | 0.71 | 0.16 | 0.33 | Generated Ligands | 0.18 | 0.13 | 486.66 | SC_12 | 0.03 | 0.22 | O=C(CC1CC2CCC(C1)N2CCc1ccc(-c2ccccn2)cc1)c1ccc(-c2ccccc2)cc1 | 8 | 33.2 | 7.34 | 0 | 3 | 37 | 1 | - | III | + | - | + | - | + | - | - | Non-required | + | - | - | - | - | + | - | + | + | - | - | + | - | - | + | + | - | - | - | 2.505939 | + | + | - | + | + | + | + | + | 0.992298 | Mitochondria | - | - | -2.82683 |
| 1.6 | -8.2 | 0.7 | -0.27 | 0.1 | Generated Ligands | -0.78 | -0.59 | 641.86 | SC_13 | -0.89 | 0.41 | Cc1ccc(CC(=O)NC(Cc2ccccn2)CC(O)C(Cc2ccccc2)NC(=O)C(C(C)C)N2CCCCC2=O)c(C(C)C)n1 | 15 | 124.52 | 4 | 3 | 9 | 47 | 1 | - | III | + | - | - | - | + | - | - | Non-required | - | - | - | - | - | - | - | + | - | - | - | + | + | + | + | + | - | - | + | 2.822253 | + | + | + | - | - | + | + | + | 0.911313 | Mitochondria | + | + | -3.14533 |
| 1.73 | -8.2 | 1.02 | -0.01 | -0.04 | Generated Ligands | -0.14 | -0.14 | 431.45 | SC_14 | -0.22 | 0.04 | CC(=O)Oc1ccccc1C(=O)Nc1ccc(C(=O)Nc2ccc(N=C(N)N)cc2)cc1 | 7 | 148.91 | 2.58 | 6 | 9 | 32 | 1 | - | III | + | - | + | - | + | - | - | Non-required | - | - | - | + | - | - | - | + | - | - | - | + | + | + | + | + | - | - | + | 2.210305 | + | + | - | - | - | - | - | + | 0.825592 | Mitochondria | - | - | -4.20646 |
| 1.12 | -8.2 | 1.44 | -0.31 | 0.06 | Generated Ligands | -0.69 | -0.55 | 628.77 | SC_15 | -0.62 | 0.46 | CC(=O)Oc1ccccc1C(=O)NC(Cc1ccccc1)C(O)CC(Cc1ccccc1)NC(=O)C(C(C)C)N1CCCNC1=O | 15 | 137.06 | 4.97 | 4 | 10 | 46 | 1 | - | III | + | - | + | - | + | - | - | Non-required | - | - | - | + | - | - | - | + | - | - | - | + | + | + | + | + | - | - | + | 2.843505 | + | + | + | - | - | + | + | + | 0.966281 | Mitochondria | + | - | -3.46273 |
| 2.61 | -8.2 | 0.45 | -0.02 | -0.07 | Generated Ligands | -0.3 | -0.31 | 496.59 | SC_16 | -0.13 | -0.13 | CC(=O)Oc1ccccc1CC(=O)c1c(CSc2ccccc2)n(C)c2c(-c3ccccc3)nnn12 | 9 | 78.51 | 5.03 | 0 | 7 | 36 | 1 | + | III | + | + | + | - | + | - | - | Non-required | - | + | + | - | - | - | + | + | + | - | - | + | + | + | + | + | - | - | + | 2.375735 | + | + | - | - | - | + | - | + | 1.06526 | Mitochondria | + | - | -3.60978 |
| 4.25 | -9.4 | 0.29 | -0.13 | -0.18 | Generated Ligands | -0.44 | -0.12 | 527.44 | SC_17 | -0.18 | -0.41 | Cc1ccc(NC(=O)c2c(CSc3ccccc3)n(C)c3cc(Br)c(O)c(O)c23)c(C)c1O | 5 | 94.72 | 6.05 | 4 | 6 | 33 | 2 | + | III | + | + | + | - | + | - | - | Non-required | + | - | - | - | - | - | + | + | + | - | - | + | + | + | + | + | + | - | + | 2.154366 | + | + | + | - | - | - | + | + | 1.045341 | Nucleus | + | + | -4.0184 |
| 0.69 | -9 | 0.68 | -2.58 | -1.93 | Generated Ligands | -3.13 | -2.95 | 804.99 | SC_18 | -3.04 | -1.25 | O=C(CC1NCCO1)NC(=O)C(Cc1ccccc1)NC(=O)C1C2CCC1CC(C(=O)NC1CC3CCC(C1)N3CCc1ccc(-c3ccccn3)cc1)C(C(=O)O)C2 | 14 | 179.06 | 3.77 | 5 | 13 | 59 | 2 | - | III | + | + | + | - | + | - | - | Non-required | - | - | - | - | - | - | - | + | - | - | - | + | + | + | - | + | - | - | + | 3.450722 | + | + | + | - | - | + | + | + | 0.885988 | Nucleus | + | - | -2.70924 |
| 0.69 | -8.6 | 0.8 | -2.25 | -1.64 | Generated Ligands | -2.92 | -2.65 | 192.98 | SC_19 | -2.71 | -1.01 | O=C(O)C[N+]([O-])(C1CC2CCC(C1)C2C(=O)NC(Cc1ccccc1)C(=O)NC(=O)CC1NCCO1)C1CC2CCC(C1)N2CCc1ccc(-c2ccccn2)cc1 | 15 | 167.03 | 2.99 | 4 | 13 | 58 | 2 | - | III | + | + | + | - | + | - | - | Non-required | - | - | - | - | - | - | - | + | - | - | - | + | + | + | + | + | - | - | + | 3.508256 | + | + | + | - | - | + | + | + | 0.866349 | Mitochondria | + | - | -2.90044 |
| 0.2 | -8.3 | 1.57 | -1.71 | -1.12 | Generated Ligands | -2.49 | -1.98 | 747.89 | SC_20 | -2.3 | -0.5 | CC(=O)Oc1ccccc1C(=O)Nc1ncccc1C(=O)NC(Cc1ccccc1)C(O)CC(Cc1ccccc1)NC(=O)C(C(C)C)N1CCCCC1=O | 17 | 167.03 | 4.81 | 4 | 12 | 55 | 2 | - | III | + | + | + | - | + | - | - | Non-required | - | - | - | + | - | - | - | + | - | - | - | + | + | + | + | + | - | - | + | 2.512695 | + | + | + | - | - | + | + | + | 0.990978 | Mitochondria | + | - | -3.52058 |

Proof of the mechanism of drug action

|  | DMSO | GC327 | Amprenavir | Asunaprevir | Atazanavir | Boceprevir | Camostat | Danoprevir | Darunavir | Gabexate | Glecaprevir | Indinavir | Lopinavir | Nilfinavir | Paritaprevir | Ritonavir | Saquinavir | Sofosbuvir | Tipranavir |
| --- | --- | --- | --- | --- | --- | --- | --- | --- | --- | --- | --- | --- | --- | --- | --- | --- | --- | --- | --- |
|  | 96.35036 | 8.394161 | 88.94009 | 78.34101 | 84.3318 | 54.8875 | 94.47005 | 70.07299 | 90.32258 | 88.01843 | 92.76163 | 83.87097 | 75.57604 | 69.12442 | 25.54745 | 82.02765 | 65.89862 | 76.4977 | 30.41475 |
|  | 104.0146 | 8.029197 | 88.01843 | 79.26267 | 88.01843 | 56.56346 | 101.8433 | 72.9927 | 93.08756 | 104.1475 | 89.30774 | 86.17512 | 76.4977 | 72.35023 | 31.38686 | 87.5576 | 67.28111 | 78.34101 | 38.70968 |
|  | 99.63504 | 5.474453 | 94.47005 | 81.10599 | 91.24424 | 59.07739 | 102.3041 | 80.65693 | 98.61751 | 90.78341 | 94.73528 | 85.25346 | 84.3318 | 70.04608 | 37.22628 | 91.24424 | 76.03687 | 81.56682 | 37.32719 |
|  |  |  |  |  |  |  |  |  |  |  |  |  |  |  |  |  |  |  |  |
|  |  |  |  |  |  |  |  |  |  |  |  |  |  |  |  |  |  |  |  |
|  |  |  |  |  |  |  |  |  |  |  |  |  |  |  |  |  |  |  |  |
| **AVG** | 100 | 7.29927 | 90.47619 | 79.56989 | 87.86482 | 56.84278 | 99.53915 | 74.57421 | 94.00922 | 94.31645 | 92.26822 | 85.09985 | 78.80185 | 70.50691 | 31.38686 | 86.94316 | 69.73887 | 78.80184 | 35.48387 |
| **SD** | 3.139537 | 1.298915 | 2.849041 | 1.149512 | 2.824081 | 1.721879 | 3.589328 | 4.463244 | 3.448531 | 7.042655 | 2.243084 | 0.946916 | 3.928329 | 1.356647 | 4.767862 | 3.787658 | 4.488983 | 2.094957 | 3.628574 |
| **SEM** | 1.814761 | 0.750818 | 1.646845 | 0.664458 | 1.632417 | 0.995306 | 2.074756 | 2.57991 | 1.993371 | 4.070899 | 1.29658 | 0.54735 | 2.27071 | 0.784189 | 2.75599 | 2.189398 | 2.594788 | 1.210958 | 2.097442 |
|  |  |  |  |  |  |  |  |  |  |  |  |  |  |  |  |  |  |  |  |
|  | DMSO | GC327 | Amprenavir | Asunaprevir | Atazanavir | Boceprevir | Camostat | Danoprevir | Darunavir | Gabexate | Glecaprevir | Indinavir | Lopinavir | Nilfinavir | Paritaprevir | Ritonavir | Saquinavir | Sofosbuvir | Tipranavir |
| **AVG** | 100 | 7.29927 | 90.47619 | 79.56989 | 87.86482 | 56.84278 | 99.53915 | 74.57421 | 94.00922 | 94.31645 | 92.26822 | 85.09985 | 78.80185 | 70.50691 | 31.38686 | 86.94316 | 69.73887 | 78.80184 | 35.48387 |
| **SEM** | 1.814761 | 0.750818 | 1.646845 | 0.664458 | 1.632417 | 0.995306 | 2.074756 | 2.57991 | 1.993371 | 4.070899 | 1.29658 | 0.54735 | 2.27071 | 0.784189 | 2.75599 | 2.189398 | 2.594788 | 1.210958 | 2.097442 |

|  | DMSO | 96.35036 | 104.0146 | 99.63504 |
| --- | --- | --- | --- | --- |
|  | GC327 | 8.394161 | 8.029197 | 5.474453 |
| 1 | Amprenavir | 88.94009 | 88.01843 | 94.47005 |
| 2 | Asunaprevir | 78.34101 | 79.26267 | 81.10599 |
| 3 | Atazanavir | 84.3318 | 88.01843 | 91.24424 |
| 4 | Boceprevir | 54.8875 | 56.56346 | 59.07739 |
| 5 | Camostat | 94.47005 | 101.8433 | 102.3041 |
| 6 | Danoprevir | 70.07299 | 72.9927 | 80.65693 |
| 7 | Darunavir | 90.32258 | 93.08756 | 98.61751 |
| 8 | Gabexate | 88.01843 | 104.1475 | 90.78341 |
| 9 | Glecaprevir | 92.76163 | 89.30774 | 94.73528 |
| 10 | Indinavir | 83.87097 | 86.17512 | 85.25346 |
| 11 | Lopinavir | 75.57604 | 76.4977 | 84.3318 |
| 12 | Nilfinavir | 69.12442 | 72.35023 | 70.04608 |
| 13 | Paritaprevir | 25.54745 | 31.38686 | 37.22628 |
| 14 | Ritonavir | 82.02765 | 87.5576 | 91.24424 |
| 15 | Saquinavir | 65.89862 | 67.28111 | 76.03687 |
| 16 | Sofosbuvir | 76.4977 | 78.34101 | 81.56682 |
| 17 | Tipranavir | 30.41475 | 38.70968 | 37.32719 |
